# A High-Throughput Sequencing Strategy for Clinical Repertoire Profiling of T Cell Receptor Beta Chain: Development and Reference Values Across Healthy Adults, Paediatrics, and Cord Blood Units

**DOI:** 10.3390/ijms26199590

**Published:** 2025-10-01

**Authors:** Emma Enrich, Mireia Antón-Iborra, Carlos Hobeich, Rut Mora-Buch, Ana Gabriela Lara-de-León, Alba Parra-Martínez, Belén Sánchez, Francisco Vidal, Pere Soler-Palacin, Francesc Rudilla

**Affiliations:** 1 Immunogenetics and Histocompatibility Laboratory, Banc de Sang i Teixits (Blood and Tissue Bank, BST), 08005 Barcelona, Catalonia, Spain; manton.extern@bst.cat; 2 Transfusional Medicine Group, Vall d’Hebron Research Institute, Universitat Autònoma de Barcelona (VHIR-UAB), 08035 Barcelona, Catalonia, Spain; chobeich@bst.cat (C.H.); rmora@bst.cat (R.M.-B.); fvidal@bst.cat (F.V.); 3 Immunology Department, Hospital Clínic, Centre de Diagnòstic Biomèdic (CDB), 08036 Barcelona, Catalonia, Spain; 4 Congenital Coagulopathy Laboratory, Banc de Sang i Teixits (Blood and Tissue Bank, BST), 08005 Barcelona, Catalonia, Spain; 5 Advanced & Cell Therapy Services, Banc de Sang i Teixits (Blood and Tissue Bank, BST), 08005 Barcelona, Catalonia, Spain; aglara@bst.cat; 6 Immunogenetics in the Autoinflammatory Response Group, Clínic Foundation for Biomedical Research-August Pi i Sunyer Biomedical Research Institute (FCRB-IDIBAPS), 08036 Barcelona, Catalonia, Spain; 7 Infection and Immunity in Pediatric Patients—Vall d’Hebron Research Institute, Vall d’Hebron Barcelona Hospital Campus, 08035 Barcelona, Catalonia, Spain; albaparramart@gmail.com (A.P.-M.); belen.sanchez@vhir.org (B.S.); pere.soler@vallhebron.cat (P.S.-P.); 8 Centro de Investigación Biomédica en Red de Enfermedades Cardiovasculares (CIBERCV), 28029 Madrid, Community of Madrid, Spain

**Keywords:** T cell receptor, next-generation sequencing analysis, molecular methodologies, molecular diagnostic

## Abstract

T cell receptor (TCR) profiling using next-generation sequencing (NGS) enables high-throughput, in-depth analysis of repertoire diversity, offering numerous clinical applications. We developed a DNA-based strategy to analyse the TCRβ-chain using NGS and established reference values for T cell repertoire characteristics in 74 healthy donors, including 44 adults, 20 paediatrics, and 10 cord blood units (CBUs). Additionally, four paediatric patients with combined immunodeficiency (CID) or severe CID (SCID) due to deleterious mutations in recombination activating genes (RAG) were analysed. The developed strategy demonstrated high specificity, reproducibility, and sensitivity, and all functional variable and joining genes were detected with minimal PCR bias. All donors had a Gaussian-like distribution of complementary-determining region 3 length, with lower presence of non-templated nucleotides and higher proportion of non-functional clonotypes in CBUs. Both CBUs and paediatrics showed greater convergence and TCRβ diversity was significantly lower in adults and donors with cytomegalovirus-positive serostatus. Finally, an analysis of paediatric patients with RAG-SCID/CID showed significantly shorter CDR3 region length and lower repertoire diversity compared to healthy paediatrics. In summary, we developed a reliable and feasible TCRβ sequencing strategy for application in the clinical setting, and established reference values that could assist in the diagnosis and monitoring of pathological conditions affecting the T cell repertoire.

## 1. Introduction

T lymphocytes play a crucial role in the adaptive immune response. Approximately 95% of circulating T cells are αβ T lymphocytes, which express α and β chains in their T cell receptors (TCRs) and recognise antigens presented by Human Leukocyte Antigen (HLA) molecules [1,2]. To recognise a broad range of antigens, the T cell repertoire, comprising the various TCRs of an individual, is highly diverse and dynamic. This diversity is enabled by the recombination of the genes that encode the variable region of α and β chains during T cell development in the thymus. In each newly developed T lymphocyte, a DNA rearrangement occurs for the variable (V), diversity (D), and joining (J) genes of the β-chain, as well as for the V and J genes of the α-chain [3,4]. The locus encoding the T cell receptor β-chain (TRB) is located on chromosome 7 and consists of 68 distinct V (TRBV), 2 D (TRBD), and 14 J (TRBJ) genes or pseudogens, whereas the α-chain locus (TRA) is located on chromosome 14 and has 61 TRAV and 61 TRAJ genes or pseudogens [5]. In addition to this recombination diversity, the insertion of non-templated (N) nucleotides occurs at the binding sites of V(D)J segments, the hypervariable complementary-determining region 3 (CDR3) [4]. This is the most variable region and the one most critical in determining the specificity of the TCR for the peptide–HLA complex [1,2]. This process, along with the combination of the α and β chains to constitute a functional TCR, makes the repertoire of a healthy individual greater than 10^8^ receptors [6].

Compared to traditional strategies for assessing the TCR repertoire, such as Sanger sequencing, spectratyping, or flow cytometry, next-generation sequencing (NGS) has enabled a more efficient and in-depth study of TCR diversity. Both DNA and RNA have been used for TCR repertoire analysis, with various strategies commercially available or published in recent years [2,7,8,9,10]. In most cases, bulk sequencing of TCR β-chain (TCRβ) is performed, as it has a higher combinatorial diversity than the α-chain due to the inclusion of the D gene segment in its recombination process [2,9].

Using NGS, the CDR3 nucleotide sequences of thousands of different clonotypes from the same individual can be identified simultaneously. As a result, it is not only possible to determine the diversity of a TCR repertoire in depth, but also to detect and monitor T cell clones of interest. In this sense, the characterisation of the TCR repertoire by NGS is being used for different clinical applications, such as to detect clonal expansions or alterations in TCR diversity in cancer, infection, autoimmunity, or immunodeficiency [1,11,12,13,14,15,16], to monitor lymphocyte reconstitution and post-transplant complications [17,18,19,20,21,22,23] and to characterise and guide T cell-based immunotherapies [2,24,25,26].

In this study, we aimed to develop a strategy for TCRβ sequencing from genomic DNA (gDNA) that would be feasible for implementation in the clinical setting. Furthermore, we considered the need to establish reference values of TCRβ repertoire characteristics in healthy individuals, including adults, paediatrics, and umbilical cord blood units (CBUs), that could assist in the diagnosis and monitoring of pathological conditions affecting the T cell repertoire. Finally, we applied the developed strategy and the reference values obtained to analyse the TCRβ repertoire in four paediatric patients with combined immunodeficiency (CID) or severe CID (SCID) due to deleterious mutations in recombination activating genes (*RAG1* and *RAG2*), which play a critical role in the V(D)J recombination process and thus in the generation of a diverse T cell repertoire [27].

## 2. Results

### 2.1. TCRβ NGS Strategy

To characterise VDJ rearrangements of the TRB loci, 51 forward and 14 reverse primers specific to TRBV and TRBJ genes, respectively, were designed (Table S1). Forward primers were located in the framework region 1 of the TRBV genes, and reverse primers in the 3′ untranslated region after TRBJ genes. In addition to the specific nucleotide sequence, each primer was designed to include a universal sequence at the 5′ end for library preparation: CS1 for TRBV primers and CS2 for TRBJ primers. Excluding universal sequences, primers had a median length of 19 pair of bases (IQR 19–20) and a melting temperature of 62 °C (IQR 60–62).

Different amplification, indexing, and purification strategies were tested to optimise library preparation and sequencing. The final optimised strategy consisted of a two-step PCR. First, gDNA amplification was performed using the designed TRBV and TRBJ primers and Platinum Taq DNA Polymerase reagents (Invitrogen, Waltham, MA, USA) (Table 1A). The amplified PCR product was directly used for library preparation, eliminating the need for gel visualisation, quantification, dilution, or any cleanup step prior to indexing. Library preparation was carried out in a second PCR, using the Single Direction Access Array Barcode Library for Illumina Sequencers 384 (Fluidigm, San Francisco, CA, USA) and FastStart High Fidelity PCR System reagents (Roche, Basilea, Switzerland) (Table 1B). The single-indexing Barcode Library is specific to each sample processed and consists of specific primers for the CS1 and CS2 universal sequences, which also includes index and Illumina adapter sequences. Both specific amplification and library preparation were carried out in a Veriti Thermal Cycler (Applied Biosystems, Waltham, MA, USA). After this two-step PCR, libraries of around 480 pairs of bases were obtained (Figure S1). Libraries from different samples were quantified using a Qubit fluorometer (Invitrogen), normalised, pooled together, and purified using the MinElute Gel Extraction kit (Qiagen). Purified libraries were adjusted to 16 pM and paired-end-sequenced in a MiSeq system using a 500-cycle MiSeq Reagent Kit v2 (Illumina, San Diego, CA, USA). Then, 10% of PhiX Control (Illumina) was added to each library before sequencing. A schematic overview of the developed strategy, outlining the required instruments and the duration of each step, is presented in Figure S2. In addition, Table S2 compares our strategy with other commercial or previously reported approaches.

Various measures were implemented to prevent and control contamination between samples. A laminar flow cabinet in a pre-PCR room was used for the preparation of both the first and second PCR. Amplified products were only handled in post-PCR rooms and all materials used in the protocol were thoroughly cleaned before use. In addition, whenever possible, replicates of each sample were processed, negative controls amplified and sequenced in each sequencing run, and empty wells left between samples on the same PCR plate. Contamination was assessed using synthetic DNA fragments representing defined TRBV–TRBJ combinations, included as positive controls in each run, and calculating the proportion of control-derived reads present in each sample. Importantly, because a median contamination rate of 0.00075% (IQR 0.00019–0.00143) was detected in our sequencing runs, samples that could share low-frequency clonotypes of interest were processed in separate runs.

### 2.2. Primer Specificity and Efficiency

**Figure 1:**
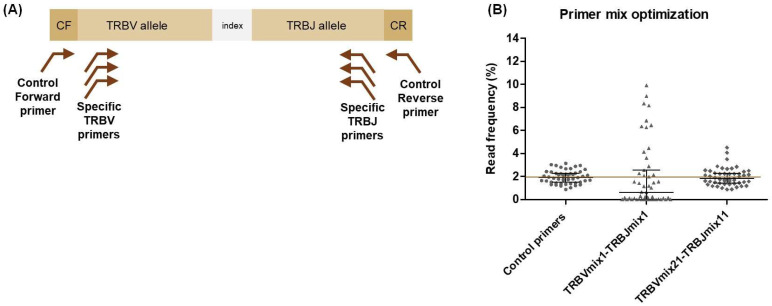
Optimisation of primer efficiency using synthetic control DNA fragments. (**A**) Each control DNA fragment was designed to contain a unique TRBV and TRBJ allele combination, along with a unique 8-nucleotide index sequence. In addition, all control DNA fragments begin and end with the same control forward and control reverse sequences, and can be amplified using the pair of control primers and the specific TRB primers. (**B**) Read frequency of each of the 51 control DNA fragments amplified with the control primer pair, with the initial TRBV1-TRBJ1 primer mix and with the optimised TRBV21-TRBJ11 primer mix. The coloured line at 1.961 (100/51) represents the expected read frequency of the 51 synthetic DNA fragments mixed in equimolar proportions. Error bars represent the median and interquartile range. CF, control forward; CR, control reverse.

Primer specificity and efficiency were evaluated and optimised to avoid multiplex PCR bias using synthetic DNA controls. Following the strategy proposed by Carlson and collaborators [28], 51 double-strand synthetic DNA fragments with different combinations of TRBV and TRBJ genes were designed. Each DNA fragment contained a TRBV allele matching the exact sequence of one of the 51 TRBV forward primers, whereas the sequence targeted by each of the 14 TRBJ primers was represented in several fragments (Figure 1A, Table S3). All fragments also contained identical control sequences at their 3′ and 5′ ends, enabling amplification with a pair of control primers (Figure 1A, Table S1). The 51 DNA fragments were pooled at equimolar concentrations and amplified with the control primers to verify the equimolarity of the pool prior to testing TRB primers specificity and efficiency (Figure S3A and Figure 1B).

TRBV primer specificity was evaluated by PCR amplification of the control DNA fragment pool using each TRBV forward primer with the control reverse primer. TRBJ primer specificity was similarly assessed using the control forward primer with each TRBJ reverse primer. In all reactions, only one TRBV- or TRBJ-specific primer was used (Figure S3B). It was found that TRBJ primers exhibited higher specificity for amplifying their target TRBJ genes compared to TRBV primers (Tables S4 and S5). Some TRBV primers could amplify TRBV genes without exact sequence homology. For example, the TRBV_FR1-05 primer showed 100% specificity for the TRBV7-1*01 allele, for which it was designed, without amplifying any other allele. In contrast, the TRBV_FR1-08 primer demonstrated 68% specificity for the allele for which it was designed (TRBV12-1*01), but could also amplify the TRBV12-3*01 allele with 28% specificity, despite a lack of exact sequence homology. This information was considered to optimise primer efficiency in the next step.

TRBV forward primers and TRBJ reverse primers were combined at equimolar concentrations to generate the TRBVmix1 and TRBJmix1, respectively. TRBV primer efficiency was evaluated by amplifying the control DNA fragment pool with TRBVmix1 and the control reverse primer, while TRBJ primer efficiency was assessed using TRBJmix1 and the control forward primer (Figure S3C). Efficiency was optimised by adjusting the proportions of individual primers within each mix. A total of 21 forward and 11 reverse mixes were tested to balance the efficiency of each primer. Since we mixed 51 synthetic DNA fragments at equimolar proportions, we expected to have a read frequency of around 1.961% per fragment following amplification and sequencing. The median read frequency obtained with the control primer pair was 1.913% (range 0.8777–3.142). When using TRBVmix1 and TRBJmix1, some DNA fragments were highly overrepresented, while others could not be amplified. The median read frequency using these mixes was 0.636%, with considerable dispersion (range 0.004–9.915). However, after optimising primer efficiency, a median read frequency of 1.82% was achieved, and the dispersion was considerably reduced (range 0.885–4.505) (Figure 1B).

Additionally, the optimised primer mix (TRBVmix21-TRBJmix11) was used to analyse six previously characterised T cell lines [29]. Two T cell lines contained only one productive rearrangement (MOLT-13 and JURKAT), whereas the others also had a second non-productive rearrangement (MOLT-3, RPMI-8402, HH, and HPB-ALL). In total, 10 rearrangements with different TRBV-TRBJ combinations were evaluated, and 100% concordance was obtained in both the determination of the CDR3 region and the assignment of TRB segments (Table S6).

### 2.3. Reproducibility and Sensitivity

**Figure 2:**
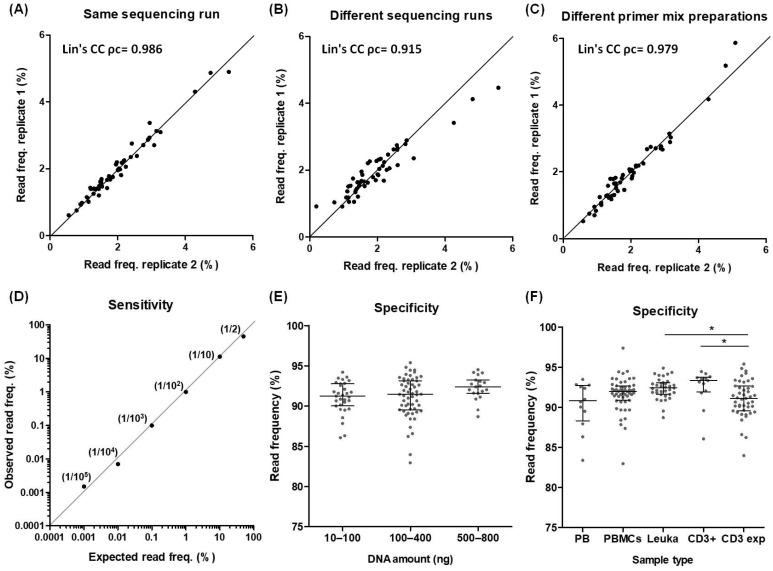
Reproducibility, sensitivity, and specificity of the designed strategy. (**A**) Correlation of the read frequency from two replicates of the same pool of control DNAs sequenced in the same sequencing run. The line represents an exact theoretical correlation between the two replicates. (**B**) Correlation of the read frequency from two replicates of the same pool of control DNAs sequenced in different sequencing runs. The line represents an exact theoretical correlation between the two replicates. (**C**) Correlation of the read frequency from two replicates of the same pool of control DNAs amplified using different preparations of the same primer mix. The line represents an exact theoretical correlation between the two replicates. (**D**) Correlation between the expected and observed read frequency from the MOLT-13 T cell line mixed at different ratios (shown next to each point) in a diverse T cell repertoire. The line represents an exact theoretical correlation between the observed and expected frequencies. (**E**) Frequency of reads specific for the TRB locus after alignment with MIXCR software (version 4.1.0) using different amounts of T cell DNA in the first PCR (10–100, *n* = 30; 100–400, *n* = 44; 500–800, *n* = 19). (**F**) Frequency of reads specific for the TRB locus after alignment with MIXCR software using different types of samples (PB, *n* = 12; PBMCs, *n* = 45; Leukapheresis, *n* = 35; enriched CD3^+^ T cells, *n* = 13; in vitro expanded T cells, *n* = 39). Concordance between two replicates was analysed using Lin’s Coefficient of Concordance (ρc). Error bars in scatter dot plots represent the median and interquartile range. Data were analysed by Kruskal–Wallis test followed by Dunn’s multiple comparison post hoc test (* *p* < 0.05). Exp, expanded; freq, frequency, leuka, leukapheresis; PB, peripheral blood; PBMCs, peripheral blood mononuclear cells.

Reproducibility was tested using replicates of the equimolar pool of synthetic control DNA fragments. High reproducibility was demonstrated after analysing replicates from the same sequencing run (Lin’s CC ρc = 0.986), from different sequencings runs (Lin’s CC ρc = 0.915), or amplified with different preparations of the same primer mix (Lin’s CC ρc = 0.979) (Figure 2A–C). Sensitivity was evaluated using the MOLT-13 T cell line mixed at known proportions with a T cell-diverse repertoire. The T cell line was detected with the expected read frequency under all ratios tested, from 1:1 to 1:10^5^ (Figure 2D).

### 2.4. Establishment of Working Samples

To determine the type of samples and the optimal T cell quantities required for obtaining accurate results with the developed strategy, DNA from peripheral blood (PB), peripheral blood mononuclear cells (PBMCs), leukapheresis, enriched CD3^+^ T cells from PB, and in vitro expanded antigen-specific T cells was used. After alignment of the sequencing reads to TRB genes, a median specificity of 91.97% (IQR 90.67–92.99) was achieved, which was greater than 80% regardless of the sample type or the initial amount of T cell DNA used in the PCR (10–800 ng corresponding to about 1500 to 120,000 T cells) (Figure 2E,F). These high alignment values obtained using MIXCR, a specialised software for TCR analysis, confirmed that the sequences were highly specific to the TRB locus. Consistently, alignment against the human genome demonstrated that more than 95% of the reads mapped to chromosome 7, specifically within the region harbouring the TRB locus [5] (Figure S4).

**Figure 3:**
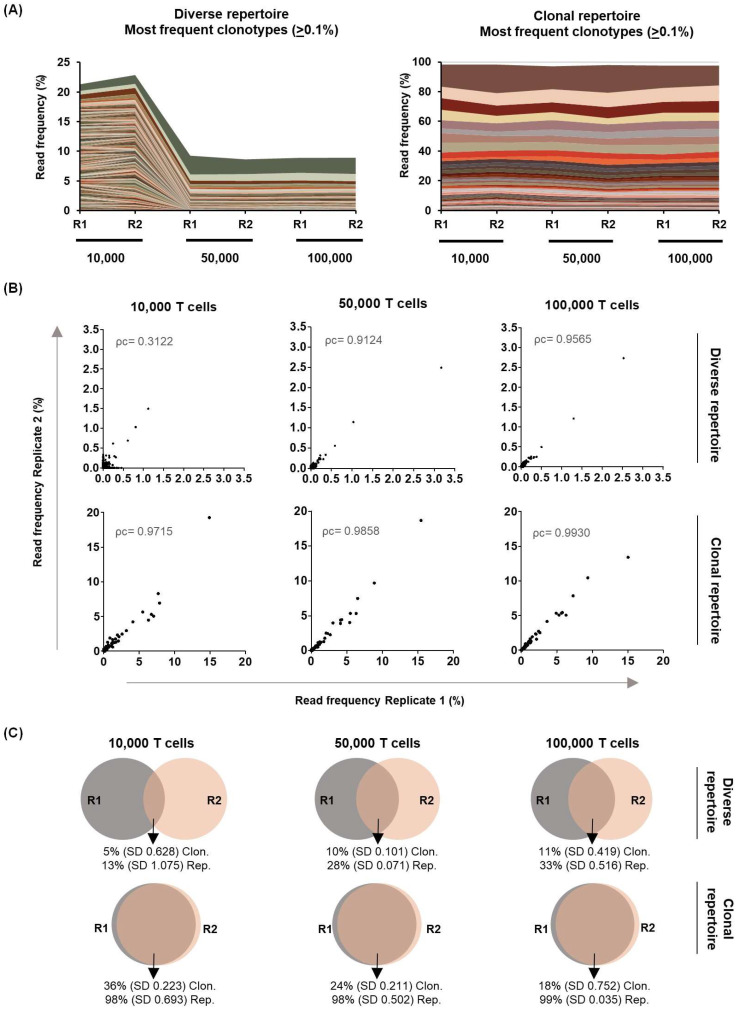
Comparative analysis of the T cell repertoire using replicates with different numbers of T cells. (**A**) Each coloured line represents a unique clonotype and its read frequency in the two replicates (R1 and R2) under the different amounts tested (10,000, 50,000, and 100,000 T cells) of the diverse and clonal repertoires. Only clonotypes with a frequency ≥ 0.1% are shown. (**B**) Correlation of the read frequency of all clonotypes between both replicates under the different T cell amounts tested. Concordance between two replicates was analysed using Lin’s Coefficient of Concordance (ρc). (**C**) Proportion of unique clonotypes (Clon) and their abundance in the repertoire (Rep) in both replicates of different samples tested.

In addition, two samples of a diverse and a clonal T cell repertoire, derived from a leukapheresis product and an in vitro T cell expansion, respectively, were sequenced in replicates using different amounts of T cells (10,000, 50,000, and 100,000). A minimum of ten reads per cell were assigned for each condition. Consistent with previous results, specificity remained greater than 80% regardless of number of T cells (Table 2). In both the clonal and diverse repertoire, the main clonotypes were detected under all conditions tested (Figure 3A and Figure S5A,B). However, the frequencies of the main clonotypes showed a slight decrease in the diverse repertoire sample in which a lower number of cells (10,000) was analysed. This condition also showed lower stability in the repertoire and the greatest discrepancy between replicates when comparing the frequencies of total clonotypes (Figure 3A,B). This discrepancy was not observed when comparing the two replicates with the lowest number of cells (10,000) in the clonal repertoire. Although the replicates of the clonal repertoire showed the greatest similarity, the use of 50,000 or 100,000 T cells also showed robustness between replicates in the diverse repertoire. Nevertheless, the number of unique clonotypes shared and their abundances between replicates were lower in the diverse compared to clonal repertoire. Only 11% of the unique clonotypes were shared between replicates in the 100,000 T cell condition, representing 33% of the diverse repertoire. In contrast, although only 18–36% of the unique clonotypes were shared in the clonal repertoire, they accounted for over 98% of the repertoire in all tested replicates (Figure 3C).

Different diversity parameters were also compared across all conditions (Table 2). On the one hand, observed diversity increased with the number of T cells sequenced, with a greater increase in the diverse repertoire studied. D50 also increased proportionally to the number of T cells analysed in the T cell-diverse repertoire, but not in the clonal repertoire. On the other hand, the normalised Shannon–Wiener index and the inverse Simpson index remained more stable across the different T cell conditions. Within each cell condition, replicates showed high similarity, indicating that initial cell number does not affect the robustness of the diversity results. A slight decrease in the inverse Simpson index was only observed in one condition of the T cell diverse repertoire (50,000 cells, replicate 1). The most frequent clonotype in this sample was slightly elevated compared to the other conditions (Figure S5A). Since the inverse Simpson’s index is primarily affected by changes in the frequencies of the major clonotypes, this could explain the observed decrease in diversity.

Finally, TRBV and TRBJ usage frequencies, along with the length distribution of the CDR3 region, were analysed. A high similarity between replicates and conditions was observed, indicating that these parameters are not affected by the initial number of T cells studied (Figure S5C–F).

### 2.5. Reference Values for TCRβ Repertoire Profiling

To obtain reference values for the T cell repertoire, a total of 74 healthy donors were sequenced, including 44 adults, 20 paediatrics, and 10 CBUs (Table 3). A median of 1.07 × 10^6^ reads (IQR 0.91 × 10^6^–1.28 × 10^6^) per sample were obtained, of which 92.18% (IQR 91.42–92.96) were specific for TRB genes. After TRB alignment, error correction, and the extraction of non-functional clonotypes, a median of 728,969 reads (IQR 624.364–840.483) per sample was used to constitute functional clonotypes.

**Figure 4:**
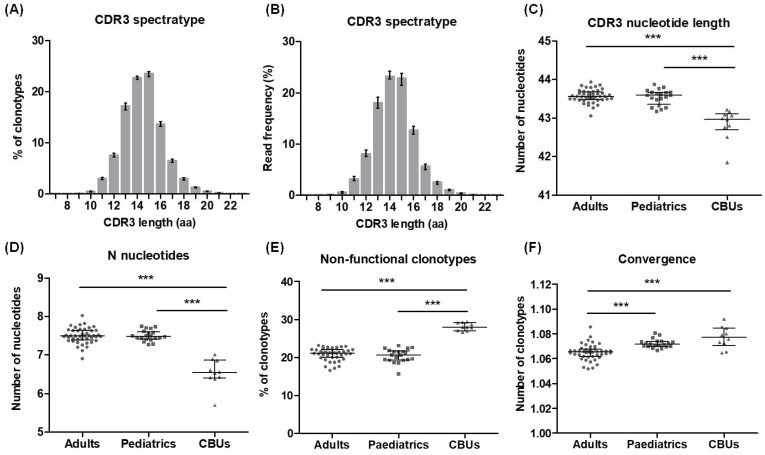
CDR3 metrics in healthy donors. (**A**) Distribution of CDR3 amino acid length across all donors (adults, paediatrics, and CBUs) calculated from unique clonotypes. Bars represent the median of clonotype frequencies with each CDR3 region length. Error bars represent the interquartile range. (**B**) Distribution of CDR3 amino acid length across all donors (adults, paediatrics, and CBUs) calculated from total sequences. Bars represent the median of clonotype frequencies with each CDR3 region length. Error bars represent the interquartile range. (**C**) Nucleotide length of the CDR3 region in adults, paediatrics, and CBUs calculated from unique clonotypes. (**D**) Number of added N nucleotides in the CDR3 region in adults, paediatrics, and CBUs calculated from total sequences. (**E**) Percentage of non-functional clonotypes relative to the total number of unique clonotypes in adults, paediatrics, and CBUs. (**F**) Mean number of unique nucleotide sequences coding for the same CDR3 amino acid sequence in adults, paediatrics, and CBUs. Error bars in scatter dot plots show median and interquartile range. Data were analysed by Kruskal–Wallis test followed by Dunn’s multiple comparison post hoc test (*** *p* < 0.001). Aa, amino acid; CBU, cord blood unit.

CDR3 region parameters, convergence, diversity, and clonality are summarised in Table 4. Although the length of the CDR3 region followed a Gaussian-like distribution for all three groups, both considering unique clonotypes or total sequences (Figure 4A,B and Figure S6), the CDR3 length and the number of N nucleotides added to this region were significantly lower in the CBU samples compared to adults and paediatrics (Figure 4C,D). In contrast, CBUs had a significantly higher proportion of non-functional clonotypes (Figure 4E). The mean number of unique nucleotide sequences coding for the same CDR3 amino acid sequence, referred to as convergence, was significantly higher in the paediatric and CBU groups compared to adults (Figure 4F).

**Figure 5:**
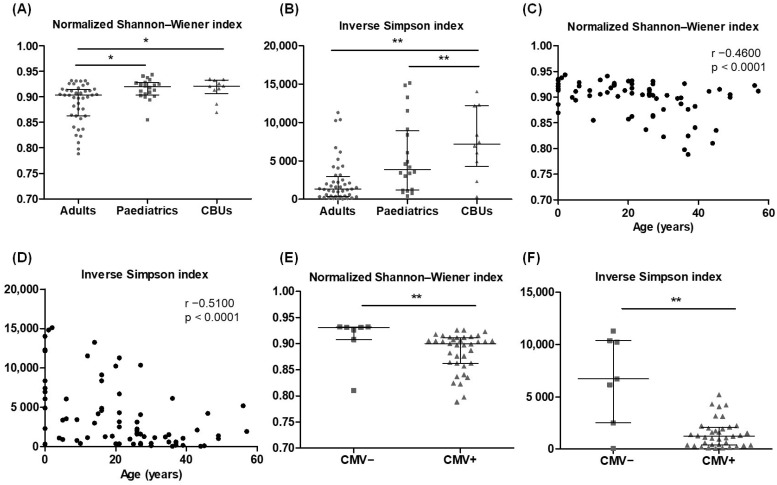
T cell repertoire diversity and clonality in healthy donors. (**A**) Normalised Shannon–Wiener index in adults, paediatrics, and CBUs. (**B**) Inverse Simpson index in adults, paediatrics, and CBUs. (**C**) Correlation of the normalised Shannon–Wiener index with the age of all donors. (**D**) Correlation of the inverse Simpson index with the age of all donors. (**E**) Normalised Shannon–Wiener index in CMV seronegative (−) and CMV seropositive (+) adult donors. (**F**) Inverse Simpson index in CMV seronegative (−) and CMV seropositive (+) adult donors. Error bars in scatter dot plots represent the median and interquartile range. Data analysed by Kruskal–Wallis test followed by Dunn’s multiple comparison post hoc test in (**A**,**B**), and by Mann–Whitney test in (**E**,**F**) (* *p* < 0.05,** *p* < 0.01). Correlation analysed using Spearman’s correlation coefficient. CBU, cord blood unit; CMV, cytomegalovirus.

The normalised Shannon–Wiener index showed significantly lower repertoire diversity in the adult samples compared to paediatrics and CBUs (Figure 5A), while both adults and paediatrics had a significantly lower inverse Simpson’s index compared to CBUs (Figure 5B). Interestingly, 13.64% of adult donors (6 of 44) had at least one clonotype with a frequency greater than 5%, whereas this was not observed in either the paediatric or CBU donors. Additionally, a statistically significant inverse correlation was detected between donor age and the two diversity indices, with a decrease in diversity and an increase in clonality at older age (Figure 5C,D). Both the normalised Shannon–Wiener index and inverse Simpson index also decreased significantly in adult donors with positive cytomegalovirus (CMV) serostatus (Figure 5E,F). In contrast, repertoire diversity was not affected by sex or the number of HLA class I and II polymorphisms in the donors within this cohort (Figure S7). Although approximately 100,000 T cells were examined per sample, the donor samples originated from different cell sources, and the number of CD3^+^ T cells could not be calculated and normalised across all samples. As shown in Table 2, this could affect the calculation of observed diversity and D50, which depend on the initial T cells studied. For this reason, these parameters were not statistically compared between groups.

**Figure 6:**
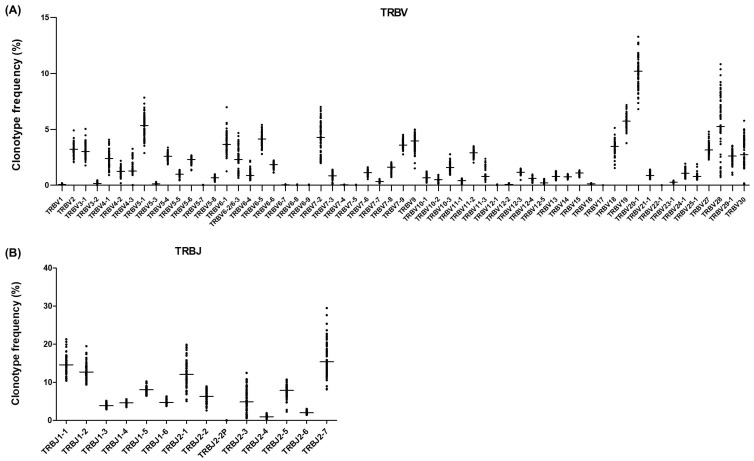
TRBV and TRBJ usage frequencies in healthy donors. (**A**) Frequency of unique clonotypes with each TRBV gene relative to the total number of unique clonotypes in each donor. All donors (adults, paediatrics, and CBUs) are presented together. The horizontal line represents the median frequency. (**B**) Frequency of unique clonotypes with each TRBJ gene relative to the total number of unique clonotypes in each donor. All donors (adults, paediatrics, and CBUs) are presented together. The horizontal line represents the median frequency.

A total of 60 TRBV genes were detected, of which 48 had functional alleles and 12 were considered Open Reading Frames (ORFs) or pseudogenes. All genes with functional alleles described in the ImMunoGeneTics (IMGT) web resource were detected with this strategy. The most frequently used TRBV genes when considering unique clonotypes were *TRBV20-1*, *TRBV19*, *TRBV5-1*, *TRBV28*, and *TRBV7-2*, with a median frequency of 10.18% (IQR 9.04–10.94), 5.73% (IQR 5.27–6.03), 5.34% (IQR 4.66–5.83), 5.26% (IQR 4.54–7.66), and 4.28% (IQR 3.04–5.16), respectively (Figure 6A). A similar pattern of frequencies was observed for adults, paediatrics, and CBUs (Figure S8). Interestingly, although similar TRBV frequencies were found when considering the number of reads for each single clonotype, it should be noted that *TRBV7-2* increased its frequency in the repertoire from 4.28% (IQR 3.04–5.16) to 10.14% (IQR 8.04–12.33) (Figure S9A,B).

As for TRBJ, the 14 genes described in IMGT were detected, 13 with functional alleles and 1 considered an ORF. Considering only unique clonotypes, the most frequently used TRBJ genes were *TRBJ2-7*, *TRBJ1-1*, *TRBJ1-2*, *TRBJ2-1*, and *TRBJ1-5*, with a median frequency of 15.39% (IQR 13.11–18.69), 14.59% (IQR 12.74–16.16), 12.76% (IQR 11.74–13.96), 12.09% (IQR 10.05–14.62), and 8.12% (IQR 7.55–8.65), respectively (Figure 6B). In this case, a similar pattern of frequencies was obtained for the three groups of donors studied, and also when frequencies were calculated considering the number of reads for each clonotype (Figure S10).

### 2.6. TCRβ Repertoire Profiling in RAG-SCID/CID Patients

The TCRβ repertoire was analysed in four paediatric patients with RAG-SCID/CID (Table 5). A median of 458,310 reads (IQR 185,832–524,216) per sample was obtained, of which 80.91% (IQR 34.59–86.51) were specific for TRB genes. After TRB alignment, error correction, and the extraction of non-functional clonotypes, a median of 334,093 reads (IQR 93,521–406,509) per sample was used to constitute functional clonotypes.

**Figure 7:**
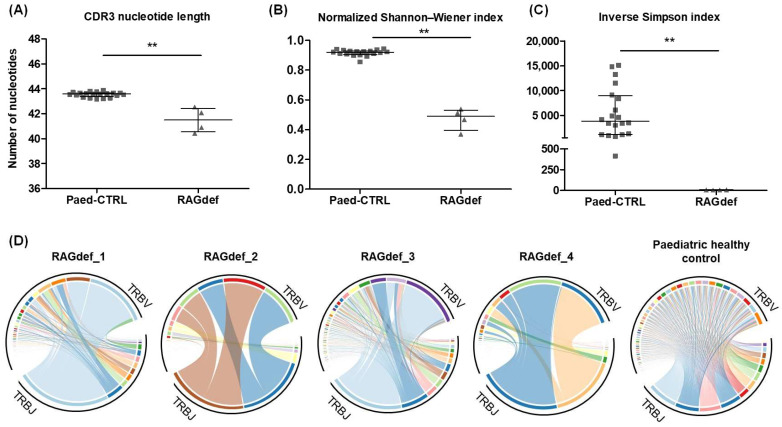
CDR3 length and repertoire diversity in patients with RAG-SCID/CID. (**A**) Nucleotide length of the CDR3 region in healthy paediatrics and patients with RAG-SCID/CID, calculated from unique clonotypes. (**B**) Normalised Shannon–Wiener index in healthy paediatrics and patients with RAG-SCID/CID. (**C**) Inverse Simpson index in healthy paediatrics and patients with RAG-SCID/CID. (**D**) TRBV and TRBJ combinations in healthy paediatrics and patients with RAG-SCID/CID considering total sequences. Each line represents a unique TRBV-TRBJ combination and its amplitude represents its abundance in the repertoire. Colors indicate different TRB genes, which are omitted for clarity. The figure highlights TRBV–TRBJ combinations overrepresented in RAG-SCID/CID patients compared with a healthy paediatric control. Error bars in scatter dot plots represent the median and interquartile range. Data analysed by the Mann–Whitney test (** *p* < 0.01). Def, deficiency; Paed-CTRL, paediatric controls; RAG, recombination activating genes; SCID/CID, severe combined immunodeficiency/combined immunodeficiency.

RAG-SCID/CID patients had a significantly shorter CDR3 region length compared to healthy paediatric controls (Figure 7A). The length distribution of the CDR3 region was analysed considering both unique clonotypes and total reads. None of the patients with RAG-SCID/CID showed a normal distribution of the T cell repertoire when total reads were considered, indicating the presence of overrepresented clonotypes (Figure S11). A median of 221 unique clonotypes (IQR 75–1197) were detected, of which only 3 (IQR 2–4) accounted for 50% of the abundance of the T cell repertoire (D50). The normalised Shannon–Wiener index and the inverse Simpson index presented a median value of 0.4899 (IQR 0.3947–0.5314) and 6.00 (IQR 4.25–7.75), respectively. Both indices were significantly lower in RAG-SCID/CID patients compared to healthy paediatric controls (Figure 7B,C). TRBV and TRBJ usage frequencies were also calculated considering both unique clonotypes and total reads. Not all patients showed substantial impairment in the use of TRB segments compared to healthy controls when considering unique clonotypes. However, an analysis considering total reads showed skewed frequencies in all of them, which is in line with the presence of overrepresented clonotypes (Figure 7D and Figure S12).

## 3. Discussion

In this work, we developed and implemented a gDNA-based strategy to characterise VDJ rearrangements of the TCRβ chain using NGS. TCR sequencing has demonstrated its broad applicability in various clinical contexts by providing a detailed view of the T cell repertoire. This technology allows the detection of alterations in the diversity of the T cell repertoire in different pathological conditions, including cancer, infections, autoimmunity, and immunodeficiency. Furthermore, TCR sequencing is valuable for monitoring lymphocyte reconstitution and complications after haematopoietic stem cell transplantation, as well as for the monitoring and development of T cell-based therapies [1,12,13,14,15,16,17,18,19,20,21,22,23,24,25,26].

Choosing gDNA rather than RNA as the starting genetic material guarantees that the amount of gDNA is proportional to the number of cells analysed and is not affected by cellular expression levels. This approach enables the determination of the relative abundance of each TCRβ clonotype within the analysed T cell repertoire [1]. Moreover, the increased stability of the gDNA and the fact that reverse transcription is not required make this strategy more practical for use, which is particularly important in clinical laboratory settings. Nevertheless, one of the main challenges of gDNA-based methodologies is the need for multiplex PCR for VDJ amplification, which can introduce substantial primer bias. In this context, and following the strategy proposed by Carlson and collaborators [28], we used a pool of synthetic DNA controls covering different combinations of TRBV-TRBJ genes to correct the efficiency of the designed specific primers. After implementing this correction, a substantial reduction in primer bias was obtained. Furthermore, all functional TRBV and TRBJ genes were detected, and high reproducibility was obtained, demonstrating the reliability of the strategy. Nonetheless, it is important to recognise the challenge of obtaining DNA controls that accurately represent the high complexity of these genes. Their high variability, along with their combinatorial diversity and the incorporation of novel nucleotides within the CDR3 region, results in a vast variety of sequences present in a PCR reaction. As a result, inherent differences in sequence composition and length can influence amplification efficiency, despite prior optimisation of primer performance.

Our strategy also demonstrated a high degree of specificity for the TRB locus, reflecting the effectiveness of protocol optimisation. Adjustments in key steps—such as the choice of polymerase, PCR annealing temperature, and the purification strategy of indexed products—were critical to prevent the presence of primer dimers or nonspecific sequences. In addition, this high specificity was achieved regardless of the type of sample tested (peripheral whole blood, PBMCs, leukapheresis products, CD3^+^ T cells, or in vitro expanded antigen-specific T cells) and the initial number of T cells used (1500–120,000). However, working with a higher number of CD3^+^ T cells allows for a more in-depth study of the T cell repertoire, which is especially important when analysing a diverse T cell repertoire. Although a high similarity in diversity metrics, TRB gene usage frequencies, and CDR3 length was observed between replicates when different numbers of T cells (10,000, 50,000, or 100,000) were used in the initial PCR, the clonotypes detected showed better reproducibility when a higher initial number of T cells was used in the diverse T cell repertoire. Normalising the initial number of T cells is also important when comparing the observed diversity and D50 between samples. However, since this is not always feasible, it should be noted that the normalised Shannon–Wiener index, the usage frequencies of TRB segments, and the length of the CDR3 region remained stable, even when different numbers of T cells were used in the initial PCR. The inverse Simpson index also shows good stability; however, it is very sensitive to small changes in the frequency of the main clonotypes.

When analysing the number of unique clonotypes shared and their abundances, we found low similarity between replicates in the diverse repertoire, even when 100,000 T cells were analysed. In this condition, only 11% of the unique clonotypes were shared between replicates, which comprised 33% of the repertoire. This reflects the extreme variability of the T cell repertoire in a healthy individual. Furthermore, it is important to consider that we can only study a very small fraction of it. An adult individual is estimated to have more than 10^8^ TCRαβ sequences in approximately 10^11^–10^12^ T cells, of which only a small fraction is expected to be found in peripheral blood [1,6]. Nevertheless, 98–99% of the abundance of the T cell repertoire was shared between replicates in all conditions tested in the clonal sample. Moreover, increasing the number of initial T cells studied resulted in a decrease in unique clonotypes shared between replicates. This suggests that using a low initial number of T cells is sufficient when studying a clonal repertoire. Increasing the number of cells and, consequently, sequencing reads not only fails to improve the characterisation of the repertoire, but also increases sequencing costs and could contribute to the accumulation of sequencing errors.

Another important challenge of TCR sequencing is the difficulty in distinguishing between real, low-frequency clonotypes and artificial sequences derived from amplification and sequencing errors [6]. In this regard, it is important to use bioinformatics programmes that perform error correction, such as MIXCR and VDJtools [30,31]. Both programs provide free command-line interface versions for academic use, with comprehensive instructions enabling use without advanced computing expertise. Additionally, contamination between samples processed and sequenced together could be a problem when studying low-frequency clonotypes. In our laboratory, various measures have been established to prevent and control contamination, including the use of laminar flow cabinets and clean material for PCR preparation and the use of negative controls and replicates when possible, as well as avoiding sequencing in the same run samples that could share low-frequency clonotypes of interest.

With our strategy, we have demonstrated a sensitivity of 10^−5^, detecting one MOLT-13 T cell in 100,000 T cells from a diverse repertoire. Although it is technically possible to increase this level of sensitivity, the limitation lies in the number of T cells studied to achieve it. To reach a sensitivity of 10^−6^, it is necessary to study at least 10^6^ T cells, which is equivalent to 66 µg of T cell gDNA. This high amount of DNA required to increase sensitivity is not easy to obtain from patient samples and is one of the limitations of using gDNA instead of mRNA [1]. On the other hand, considering a minimum of 10 reads per cell, more than 10^7^ reads per sample would be required to achieve a sensitivity of 10^−6^, which substantially increases sequencing costs. Other commercial platforms for TCRβ repertoire analysis also operate at a scale of up to 10^5^ T cells. For instance, Adaptive Biotechnologies’ TCRB assay specifies input ranges of 1000–600,000 T cells in its sample preparation guidelines, while Invivoscribe’s LymphoTrack recommends similar sensitivity (up to 10^5^) for applications such as minimal residual disease detection.

For successful implementation of this strategy in our clinical laboratory, it is crucial to have reference T cell repertoire values from healthy individuals. Considering that the diversity of the T cell repertoire changes throughout life, we analysed samples from a total of 74 healthy donors, of which 44 were adult donors, 20 were paediatric donors, and 10 were CBUs. Regarding CDR3 region metrics, all donor groups showed a Gaussian-like distribution of CDR3 length, which is expected to be found in healthy individuals. However, CBUs showed a significantly shorter CDR3 region length, likely due to a significantly reduced addition of N nucleotides. Similar results were reported by Britanova and collaborators, who performed TCRβ sequencing in eight CBUs and 65 healthy individuals of different ages [32]. Accordingly, a lower activity of terminal deoxynucleotidyl transferase (TdT), which is responsible for the addition of N nucleotides during V(D)J rearrangement, was described during the foetal period [33,34,35]. In addition, a higher proportion of non-functional clonotypes were observed in CBUs, which was also described by Britanova and collaborators using an RNA-based strategy [32].

Both CBU and paediatric donors showed increased convergence compared to adults, reflecting a greater presence of T cells sharing the same CDR3 amino acid sequence but encoded by different nucleotide sequences due to codon degeneracy. The decrease in convergence at older age may result from a wider exposure to multiple antigens, which contributes to the clonal expansion of antigen-specific T cells, and by a non-uniform peripheral homeostatic proliferation of naïve T cells [6,32,36]. The normalised Shannon–Wiener index showed a significantly higher repertoire diversity in CBUs and paediatrics compared to adults. This correlation of repertoire diversity with age has been widely described in the literature and is due to a decrease in the production of naïve T cells and the accumulation of clonally expanded memory T cells [32,37,38,39,40]. In addition, adult donors showed a significantly higher inverse Simpson index, which is more sensitive to the presence of high-frequency clonotypes. Accordingly, clonotypes with frequencies higher than 5% were only detected in the adult group. Zhuo and collaborators studied a cohort of 582 healthy donors and also reported that the presence of large clones (>1% of total sequences) increased with age [40]. Considering that age-related changes in T cell repertoire diversity affect CD4^+^ and CD8^+^ T cells differently [39], a limitation of our results is that the two populations were not studied independently. Furthermore, it should also be noted that the adult cohort studied was between 19 and 57 years of age. Therefore, it would be valuable to establish reference values for the T cell repertoire in older individuals, such as those over 60 years, which is important for applying this strategy in clinical settings.

Krishna and collaborators reported that TCR repertoire diversity was associated with CMV infection in a cohort of 666 individuals [38]. Consistent with their results, we observed that CMV-seropositive individuals had significantly reduced repertoire diversity compared to CMV seronegative individuals. The authors also reported higher TCR repertoire diversity in individuals fully heterozygous at HLA class I loci compared to homozygous individuals, but this association was limited to the CMV seronegative cohort, indicating a dominant influence of CMV infection on repertoire diversity. In our study, we were unable to assess the effect of HLA independently of CMV status due only 15.91% of the adult donors (*n* = 7) were CMV-seronegative, which likely accounts for the absence of a significant association in our data. Regarding T cell repertoire diversity and sex, although other studies have suggested different dynamics of T cell repertoire diversity between males and females at different ages, we did not observe an association between these two variables in our cohort [32,38,41].

All TRBV and TRBJ genes with functional alleles could be detected using the developed strategy. As is well established in the literature, not all genes participate in the same frequency to constitute VDJ rearrangements, with *TRBV20-1*, *TRBV19*, *TRBJ2-7*, and *TRBJ1-1* being the most represented genes. On the other hand, we have also detected genes that contribute minimally to VDJ rearrangements. Most of them are pseudogenes, but we have also identified genes with functional alleles, such as *TRBV6-8*, *TRBV6-9*, *TRBV7-4*, and *TRBV16*, all of them reported at similar frequencies. Overall, the frequencies obtained are similar to those described in other studies with healthy donors [42,43]. Of note, the study by Ma and collaborators [42] employed a similar gDNA-based commercial strategy using the ImmunoSEQ assay from Adaptive Biotechnologies.

Finally, the developed strategy was applied to characterise the TCRβ repertoire in RAG-SCID/CID patients. A shorter CDR3 region length and a significantly lower repertoire diversity was observed in these patients compared to healthy paediatric donors, with only a few clonotypes covering more than 50% of the repertoire abundance. These results were in line with previously reported data [15,16]. Importantly, the repertoire data from the cohort of healthy paediatric donors provided insight into the degree of TCRβ repertoire impairment in these patients.

The results obtained, aligning with previously reported data in the literature, support the reliability of the developed strategy and highlight the importance of the established reference values. Thus, our technique holds substantial potential in various clinical contexts by providing a detailed view of the T cell repertoire and offering important applications for diagnosis, treatment monitoring, and therapy development.

## 4. Materials and Methods

### 4.1. Samples and Ethics Committee

The samples used for the development and validation of the strategy were obtained from PB, PBMCs, CD3^+^ cells, or in vitro expanded antigen-specific T cell products. These samples were obtained from anonymised blood donors from the BioBanc of the Blood and Tissue Bank (BST) or the ReDoCel registry. The ReDoCel registry was established in 2017 at our institution with the aim of obtaining well-characterised donors in terms of HLA genotype and viral reactivity for virus-specific T cell production [44]. Reference values of the TCR repertoire were obtained from PBMCs or excess leukapheresis samples from 44 ReDoCel adult donors, from PBMCs from 20 paediatric donors, and from mononuclear cells (MNCs) from 10 CBU from the BioBanc. Finally, 4 paediatric patients with RAG-SCID/CID from the Vall d’Hebron Hospital were included in the study. All donors signed an informed consent form, and the use of the samples was approved by the Vall d’Hebron Ethics Committee (PR(BS)431/2022 and PR(AMI) 515/2022). Finally, DNA from six T cell lines (MOLT-3, MOLT-13, RPMI-8402, JURKAT, HH, and HPB-ALL) was acquired from the DSMZ Leibniz Institute.

The PBMCs and cord blood MNCs were isolated by density gradient centrifugation using Lymphoprep^TM^ and SepMate^TM^ isolation tubes (Stem Cell Technologies, Vancouver, BC, Canada). For CD3^+^ selection, the PBMCs were magnetically labelled with CD3 MicroBeads and purified using the autoMACS^®^ separator (Milteny Biotech, Bergisch Gladbach, Germany). Expanded T cells were obtained after stimulation with specific viral antigens and cytokines. Genomic DNA extraction was performed using the QIAamp DNA Blood Mini Kit (Qiagen, Hilden, Germany). Amplified cell counts were extrapolated based on the DNA concentration and the weight of a diploid genome (~6.6 pg).

### 4.2. CD3^+^ T Cell Quantification Using Flow Cytometry

For the quantification of CD3^+^ T cells, samples from PB, PBMCs, CD3^+^ selection, or in vitro-expanded products were labelled with CD45-FITC (J33), CD3-PC7 (UCHT1) and 7-AAD (Beckman Coulter, Brea, CA, USA). Perfect-Count Microspheres (Cytognos, Salamanca, Spain) were also used for absolute quantification. Additionally, VersaLyse Lysing Solution (Beckman Coulter) was employed, when necessary, to lyse red blood cells prior to cell acquisition using Navios or CytoFLEX flow cytometers (Beckman Coulter).

### 4.3. Primer Design and Optimisation

To design specific primers for amplifying VDJ rearrangements of the TRB loci, all TRBV and TRBJ genes and alleles were downloaded from GenBank and IMGT/GENE-DB, and aligned using the QIAGEN CLC Genomics Workbench (Qiagen). All primers were designed to have similar melting temperatures and lengths.

To evaluate primer specificity and efficiency and following the strategy proposed by Carlson and collaborators [28], double-strand synthetic DNA fragments were designed to contain different known combinations of TRBV and TRBJ genes and were acquired as gBlocks Gene Fragments (Integrated DNA Technologies, Coralville, IA, USA).

### 4.4. TCRβ Repertoire Profiling in Healthy Donors and Patients with RAG-SCID/CID

Samples from a total of 74 healthy donors were sequenced, of which 44 were leukapheresis or PBMCs from adult donors, 20 PBMCS from paediatric donors, and 10 MNCs from CBUs. Of the 35 leukapheresis samples analysed, total lymphocyte count data were obtained from all samples, whereas CD3^+^ T cell count data, calculated by flow cytometry, were obtained from 17 samples. The CD3^+^/total lymphocyte ratio (mean 0.73, SD 0.07) was used to estimate the percentage of CD3^+^ T cells in the remaining 18 leukapheresis samples, and approximately 100,000 T cells were sequenced per sample. For PBMC and MNC samples, no data on CD3^+^ cell quantification were available. Considering that 45–70% of PBMCs are T cells, approximately 200,000 total cells were sequenced per sample. In addition, 4 patients with RAG-SCID/CID were included in the study. In this case, a median of 62,500 PBMCs (IQR 46,452–93,934) were studied per sample.

### 4.5. Sequencing Analysis

Sequencing reads obtained from the amplification of synthetic DNA controls were analysed using the QIAGEN CLC Genomics Workbench (Qiagen), aligning them with the designed reference sequences. FASTQ from all other samples were analysed directly using MIXCR (version 4.1.0) (MiLaboratories, San Francisco, CA, USA) [30,31] and VDJtools (version 1.2.1) bioinformatics software, without any additional preprocessing. MIXCR performed the primary analysis, including the alignment of raw sequencing reads, clonotype assembly, and the correction of PCR and sequencing errors. Secondary analysis was performed only for productive clonotypes using both MIXCR and VDJtools. CDR3 metrics were presented as CDR3 length and the number of added non-templated (N) nucleotides. Convergence, defined as the mean number of unique CDR3 nucleotide sequences that encode the same CDR3 amino acid sequence, was also determined. To evaluate diversity, normalised Shannon–Wiener and inverse Simpson indices, observed diversity, and D50 were calculated. Observed diversity represents the number of unique clonotypes, while D50 is the number of unique clonotypes, ordered by decreasing frequency, that account for 50% of the total repertoire abundance. For the calculation of all diversity parameters, the samples were normalised according to read counts. Frequencies of TRBV and TRBJ gene usage were also calculated, considering either the number of reads or only unique clonotypes.

### 4.6. Statistical Analysis

The D’Agostino–Pearson test was used to assess Gaussian distribution. To analyse quantitative variables from different groups, Mann–Whitney, Kruskal–Wallis, and Dunn’s multiple comparison tests were used. Correlation between two quantitative variables was determined by Spearman’s correlation coefficient. Statistical tests were conducted using GraphPad Prism (version 5.03). Lin’s Coefficient of Concordance (ρc) was used to evaluate the agreement between two replicates measurements using StatsToDo (https://www.statstodo.com). Statistical significance was considered at a *p*-value ≤ 0.05.

## Figures and Tables

**Table 1:** Amplification and library preparation protocol.

(A) First PCR—TRB Specific Amplification					
PCR Reaction (Final Volume: 25 µL)			Thermocycler Program		
Reagent	Stock Concentration	Volume/Reaction	Cycles	Temperature	Time
PCR buffer without MgCl_2_	10X	2.5 µL	1	50 °C	2 min
MgCl_2_	50 mM	0.75 µL		70 °C	20 min
dNTPs	10 mM	0.5 µL		95 °C	10 min
TRBV primer mix	3 µM	5 µL	35	95 °C	15 s
TRBJ primer mix	3.4 µM	5 µL		62 °C	15 s
Platinum Taq DNA Polymerase		0.1 µL		72 °C	30 s
H_2_O		6.15 µL	1	72 °C	3 min
DNA		5 µL			
(B) Second PCR—Library Preparation					
PCR Reaction (Final Volume: 20 µL)			Thermocycler Program		
Reagent	Stock Concentration	Volume/Reaction	Cycles	Temperature	Time
FastStart HF Buffer without MgCl_2_	10X	2 µL	1	95 °C	10 min
MgCl_2_	25 mM	3.6 µL	15	95 °C	15 s
DMSO		1 µL		60 °C	30 s
PCR Nucleotide Mix	10 mM	0.4 µL		72 °C	1 min
FastStart HF Enzyme		0.2 µL	1	72 °C	3 min
H_2_O		7.8 µL			
Barcode	2 µM	4 µL			
First PCR product		1 µL			

DMSO, Dimethyl sulfoxide; HF, high fidelity; PCR, polymerase chain reaction; TRB, T cell receptor β-chain locus

**Table 2:** Specificity and diversity according to the number of T cells in a clonal and a diverse T cell repertoire.

	T Cells Analysed	Replicate	Total Reads	TRB Alignment	Observed Diversity	D50	Normalised Shannon–Wiener Index	Inverse Simpson Index
Diverse T cell repertoire	10,000	1	108,297	88%	4718	527	0.9074	1094.18
		2	160,836	90%	5696	519	0.8900	933.81
	50,000	1	833,190	92%	33192	2981	0.8936	760.16
		2	704,224	91%	33686	3086	0.8972	1048.24
	100,000	1	1,085,346	93%	55812	4900	0.8958	1028.91
		2	1,079,824	93%	59041	5057	0.8942	951.34
Clonal T cell repertoire	10,000	1	134,485	90%	224	6	0.6245	17.25
		2	138,687	89%	226	6	0.6233	15.05
	50,000	1	763,219	92%	562	7	0.5506	17.81
		2	767,594	92%	569	6	0.5269	14.75
	100,000	1	1,186,708	91%	958	7	0.4996	17.15
		2	1,080,508	91%	902	7	0.5075	18.02

**Table 3:** Healthy donors to obtain T cell repertoire reference values.

	Adults		Paediatrics	CBUs
Type of sample	PBMCs	Leukapheresis	PBMCs	MNCs
N	9	35	20	10
Age (years)	28 (19–57)		11 (1–17)	0
Sex	65.91% F, 34.09% M		50% F, 50% M	ND
IgG CMV	15.91% Neg, 84.09% Pos		ND	ND
HLA class I (HLA-A, -B, -C)	23% ≥ 1 HM loci, 77% HT loci		ND	ND
HLA class II (HLA-DRB1, -DQB1)	25% ≥ 1 HM loci, 75% HT loci		ND	ND

CMV, cytomegalovirus; F, female; HM, homozygous; HT, heterozygous; M, male; MNCs, mononuclear cells; Neg, negative; ND, not determined; Pos, positive; PBMCs, peripheral blood mononuclear cells. Age is presented as the median and range.

**Table 4:** CDR3 region parameters, convergence, diversity, and clonality in healthy donors.

	Adults	Paediatrics	CBUs	*p*-Value
CDR3 nucleotide length	43.56 (43.48–43.68)	43.59 (43.36–43.67)	42.97 (42.70–43.11)	0.0001
N-nucleotides added to CDR3	7.50 (7.39–7.64)	7.48 (7.42–7.61)	6.55 (6.41–6.87)	<0.0001
Non-functional clonotypes (%)	21.16 (20.02–22.14)	20.64 (19.32–21.78)	28.01 (27.07–29.15)	<0.0001
Convergence	1.065 (1.062–1.068)	1.072 (1.070–1.074)	1.077 (1.071–1.085)	<0.0001
Observed diversity	43,639 (37,303–47,255)	45,242 (39,992–52,400)	28,250 (22,106–40,594)	ND
D50	3811 (3081–4903)	4483 (3803–6053)	2861 (2015–4827)	ND
Norm. Shannon–Wiener index	0.9037 (0.8631–0.9147)	0.9199 (0.9036–0.9282)	0.9214 (0.9064–0.9331)	0.0021
Inverse Simpson index	1350 (375–2976)	3860 (1175–8939)	7190 (4257–12,196)	0.0002

Values presented as median and IQR. CBU, cord blood units; IQR, interquartile range; ND, not determined; N-nucleotides, non-templated nucleotides; Norm, normalised.

**Table 5:** Demographic data and clinical characteristics of RAG-SCID/CID patients.

	RAGdef _1	RAGdef_2	RAGdef_3	RAGdef_4
Diagnostic (gene affected)	CID (*RAG1*)	SCID (*RAG2*)	CID (*RAG1*)	SCID (*RAG2*)
Age (months)	24	2.5	48	2
Sex	M	M	M	M
Lymphocytes (×10^9^/L)	13.90	0.60	1.70	1.10
CD3^+^ cells (×10^9^/L)	13.73	0.02	0.87	0.52
CD4^+^ cells (×10^9^/L)	0.94	0	0.17	0.47
CD8^+^ cells (×10^9^/L)	7.20	0.04	0.25	0.01

Def, deficiency; RAG, recombination activating genes; SCID/CID, severe combined immunodeficiency/combined immunodeficiency.

## Data Availability

The data presented in this study are available on request from the corresponding author due to ethical reasons.

## Supplementary Materials

The following supporting information can be downloaded at https://www.mdpi.com/article/10.3390/ijms26199590/s1.

## Abbreviations

The following abbreviations are used in this manuscript:

BST	Blood and Tissue Bank
CBU	Umbilical cord blood unit
CDR3	Complementary-determining region 3
CID	Combined immunodeficiency
CMV	Cytomegalovirus
D	Diversity
FR	Framework region
gDNA	Genomic DNA
HLA	Human Leukocyte Antigen
IMGT	ImMunoGeneTics
J	Joining
MNC	Mononuclear cell
mRNA	Messenger RNA
NGS	Next-generation sequencing
ORF	Open Reading Frame
PB	Peripheral blood
PBMCs	Peripheral blood mononuclear cells
RAG	Recombination activating gene
SCID	Severe combined immunodeficiency
TCR	T cell receptor
TCRβ	T cell receptor β-chain
TdT	Terminal deoxynucleotidyl transferase
TRAD	T cell receptor alpha diversity gene
TRAJ	T cell receptor alpha joining gene
TRAV	T cell receptor alpha variable gene
TRB	T cell receptor gene
TRBD	T cell receptor beta diversity gene
TRBJ	T cell receptor beta joining gene
TRBV	T cell receptor beta variable gene
UTR	Untranslated region
V	Variable

## References

[1] Mazzotti L., Gaimari A., Bravaccini S., Maltoni R., Cerchione C., Juan M., Azucena-Gonzalez E., Pasetto A., Nascimento D., Ancarani V. (2022). T-Cell Receptor Repertoire Sequencing and Its Applications: Focus on Infectious Diseases and Cancer. Int. J. Mol. Sci..

[2] Frank M.L., Lu K., Erdogan C., Han Y., Hu J., Wang T., Heymach J.V., Zhang J., Reuben A. (2023). T-Cell Receptor Repertoire Sequencing in the Era of Cancer Immunotherapy. Clin. Cancer Res..

[3] Clambey E.T., Davenport B., Kappler J.W., Marrack P., Homann D. (2014). Molecules in medicine mini review: The αβ T cell receptor. J. Mol. Med..

[4] Turner S.J., Doherty P.C., McCluskey J., Rossjohn J. (2006). Structural determinants of T-cell receptor bias in immunity. Nat. Rev. Immunol..

[5] Lefranc M.P., Giudicelli V., Duroux P., Jabado-Michaloud J., Folch G., Aouinti S., Carillon E., Duvergey H., Houles A., Paysan-Lafosse T. (2015). IMGT^®^, the international ImMunoGeneTics information system^®^ 25 years on. Nucleic Acids Res..

[6] Weng N.P. (2023). Numbers and odds: TCR repertoire size and its age changes impacting on T cell functions. Semin. Immunol..

[7] Nielsen S.C.A., Boyd S.D. (2018). Human adaptive immune receptor repertoire analysis-Past, present, and future. Immunol. Rev..

[8] Six A., Mariotti-Ferrandiz M.E., Chaara W., Magadan S., Pham H.P., Lefranc M.P., Mora T., Thomas-Vaslin V., Walczak A.M., Boudinot P. (2013). The past, present, and future of immune repertoire biology—The rise of next-generation repertoire analysis. Front. Immunol..

[9] Rosati E., Dowds C.M., Liaskou E., Henriksen E.K.K., Karlsen T.H., Franke A. (2017). Overview of methodologies for T-cell receptor repertoire analysis. BMC Biotechnol..

[10] Brüggemann M., Kotrová M., Knecht H., Bartram J., Boudjogrha M., Bystry V., Fazio G., Fronková E., Giraud M., Grioni A. (2019). Standardized next-generation sequencing of immunoglobulin and T-cell receptor gene recombinations for MRD marker identification in acute lymphoblastic leukaemia; a EuroClonality-NGS validation study. Leukemia.

[11] Medina A., Puig N., Flores-Montero J., Jimenez C., Sarasquete M.E., Garcia-Alvarez M., Prieto-Conde I., Chillon C., Alcoceba M., Gutierrez N.C. (2020). Comparison of next-generation sequencing (NGS) and next-generation flow (NGF) for minimal residual disease (MRD) assessment in multiple myeloma. Blood Cancer J..

[12] Mitchell A.M., Michels A.W. (2020). T cell receptor sequencing in autoimmunity. J. Life Sci..

[13] Nakayama M., Michels A.W. (2021). Using the T Cell Receptor as a Biomarker in Type 1 Diabetes. Front. Immunol..

[14] Farmanbar A., Kneller R., Firouzi S. (2019). RNA sequencing identifies clonal structure of T-cell repertoires in patients with adult T-cell leukemia/lymphoma. NPJ Genomic Med..

[15] Lee Y.N., Frugoni F., Dobbs K., Tirosh I., Du L., Ververs F.A., Ru H., Ott de Bruin L., Adeli M., Bleesing J.H. (2016). Characterization of T and B cell repertoire diversity in patients with RAG deficiency. Sci. Immunol..

[16] Fang M., Su Z., Abolhassani H., Zhang W., Jiang C., Cheng B., Luo L., Wu J., Wang S., Lin L. (2022). T Cell Repertoire Abnormality in Immunodeficiency Patients with DNA Repair and Methylation Defects. J. Clin. Immunol..

[17] Pagliuca S., Gurnari C., Hong S., Zhao R., Kongkiatkamon S., Terkawi L., Zawit M., Guan Y., Awada H., Kishtagari A. (2021). Clinical and basic implications of dynamic T cell receptor clonotyping in hematopoietic cell transplantation. JCI Insight.

[18] Schultze-Florey C.R., Kuhlmann L., Raha S., Barros-Martins J., Odak I., Tan L., Xiao Y., Ravens S., Hambach L., Venturini L. (2021). Clonal expansion of CD8+ T cells reflects graft-versus-leukemia activity and precedes durable remission following DLI. Blood Adv..

[19] Goel M., Eugster A., Schetelig J., Bonifacio E., Bornhäuser M., Link-Rachner C.S. (2023). Potential of TCR sequencing in graft-versus-host disease. Bone Marrow Transplant..

[20] Leick M., Gittelman R.M., Yusko E., Sanders C., Robins H., DeFilipp Z., Nikiforow S., Ritz J., Chen Y. (2020). T Cell Clonal Dynamics Determined by High-Resolution TCR-β Sequencing in Recipients after Allogeneic Hematopoietic Cell Transplantation. Biol. Blood Marrow Transplant..

[21] Kuzich J.A., Kankanige Y., Guinto J., Ryland G., McBean M., Wong E., Koldej R., Collins J., Westerman D., Ritchie D. (2021). T cell receptor beta locus sequencing early post-allogeneic stem cell transplant identifies patients at risk of initial and recurrent cytomegalovirus infection. Bone Marrow Transplant..

[22] Milano F., Emerson R.O., Salit R., Guthrie K.A., Thur L.A., Dahlberg A., Robins H.S., Delaney C. (2020). Impact of T Cell Repertoire Diversity on Mortality Following Cord Blood Transplantation. Front. Oncol..

[23] Buhler S., Bettens F., Dantin C., Ferrari-Lacraz S., Ansari M., Mamez A.C., Masouridi-Levrat S., Chalandon Y., Villard J. (2020). Genetic T-cell receptor diversity at 1 year following allogeneic hematopoietic stem cell transplantation. Leukemia.

[24] Huisman W., Roex M.C.J., Hageman L., Koster E.A.S., Veld S.A.J., Hoogstraten C., van Balen P., van Egmond H.M., van Bergen C.A.M., Einsele H. (2023). Tracking the progeny of adoptively transferred virus-specific T cells in patients posttransplant using TCR sequencing. Blood Adv..

[25] Keller M.D., Darko S., Lang H., Ransier A., Lazarski C.A., Wang Y., Hanley P.J., Davila B.J., Heimall J.R., Ambinder R.F. (2019). T-cell receptor sequencing demonstrates persistence of virus-specific T cells after antiviral immunotherapy. Br. J. Haematol..

[26] Mora-Buch R., Tomás-Marín M., Enrich E., Antón-Iborra M., Martorell L., Valdivia E., Lara-de-León A.G., Aran G., Piron M., Querol S. (2023). Virus-Specific T Cells From Cryopreserved Blood During an Emergent Virus Outbreak for a Potential Off-the-Shelf Therapy. Transplant. Cell Ther..

[27] Delmonte O.M., Villa A., Notarangelo L.D. (2020). Immune dysregulation in patients with RAG deficiency and other forms of combined immune deficiency. Blood.

[28] Carlson C.S., Emerson R.O., Sherwood A.M., Desmarais C., Chung M.W., Parsons J.M., Steen M.S., LaMadrid-Herrmannsfeldt M.A., Williamson D.W., Livingston R.J. (2013). Using synthetic templates to design an unbiased multiplex PCR assay. Nat. Commun..

[29] Tan K.T., Ding L.W., Sun Q.Y., Lao Z.T., Chien W., Ren X., Xiao J.F., Loh X.Y., Xu L., Lill M. (2018). Profiling the B/T cell receptor repertoire of lymphocyte derived cell lines. BMC Cancer.

[30] Bolotin D.A., Poslavsky S., Mitrophanov I., Shugay M., Mamedov I.Z., Putintseva E.V., Chudakov D.M. (2015). MiXCR: Software for comprehensive adaptive immunity profiling. Nat. Methods.

[31] Bolotin D.A., Poslavsky S., Davydov A.N., Frenkel F.E., Fanchi L., Zolotareva O.I., Hemmers S., Putintseva E.V., Obraztsova A.S., Shugay M. (2017). Antigen receptor repertoire profiling from RNA-seq data. Nat. Biotechnol..

[32] Britanova O.V., Shugay M., Merzlyak E.M., Staroverov D.B., Putintseva E.V., Turchaninova M.A., Mamedov I.Z., Pogorelyy M.V., Bolotin D.A., Izraelson M. (2016). Dynamics of Individual T Cell Repertoires: From Cord Blood to Centenarians. J. Immunol..

[33] Yang L., Jin R., Lu D., Ge Q. (2020). T cell Tolerance in Early Life. Front. Immunol..

[34] George J.F., Schroeder H.W. (1992). Developmental regulation of D beta reading frame and junctional diversity in T cell receptor-beta transcripts from human thymus. J. Immunol..

[35] Zemlin M., Schelonka R.L., Bauer K., Schroeder H.W. (2002). Regulation and chance in the ontogeny of B and T cell antigen receptor repertoires. Immunol. Res..

[36] Rudd B.D., Venturi V., Li G., Samadder P., Ertelt J.M., Way S.S., Davenport M.P., Nikolich-Zugich J. (2011). Nonrandom attrition of the naive CD8+ T-cell pool with aging governed by T-cell receptor:pMHC interactions. Proc. Natl. Acad. Sci. USA.

[37] Britanova O.V., Putintseva E.V., Shugay M., Merzlyak E.M., Turchaninova M.A., Staroverov D.B., Bolotin D.A., Lukyanov S., Bogdanova E.A., Mamedov I.Z. (2014). Age-related decrease in TCR repertoire diversity measured with deep and normalized sequence profiling. J. Immunol..

[38] Krishna C., Chowell D., Gönen M., Elhanati Y., Chan T.A. (2020). Genetic and environmental determinants of human TCR repertoire diversity. Immun. Ageing.

[39] Sun X., Nguyen T., Achour A., Ko A., Cifello J., Ling C., Sharma J., Hiroi T., Zhang Y., Chia C.W. (2022). Longitudinal analysis reveals age-related changes in the T cell receptor repertoire of human T cell subsets. J. Clin. Investig..

[40] Zhuo Y., Yang X., Shuai P., Yang L., Wen X., Zhong X., Yang S., Xu S., Liu Y., Zhang Z. (2022). Evaluation and comparison of adaptive immunity through analyzing the diversities and clonalities of T-cell receptor repertoires in the peripheral blood. Front. Immunol..

[41] Gong M., Li X., Zheng A., Xu H., Xie S., Yan R., Wu H., Wang Z. (2021). Age-related changes in the TRB and IGH repertoires in healthy adult males and females. Immunol. Lett..

[42] Ma L., Yang L., Shi B., He X., Peng A., Li Y., Zhang T., Sun S., Ma R., Yao X. (2016). Analyzing the CDR3 Repertoire with respect to TCR-Beta Chain V-D-J and V-J Rearrangements in Peripheral T Cells using HTS. Sci. Rep..

[43] Hou X., Wang M., Lu C., Xie Q., Cui G., Chen J., Du Y., Dai Y., Diao H. (2016). Analysis of the Repertoire Features of TCR Beta Chain CDR3 in Human by High-Throughput Sequencing. Cell Physiol. Biochem..

[44] Rudilla F., Carrasco-Benso M.P., Pasamar H., López-Montañés M., Andrés-Rozas M., Tomás-Marín M., Company D., Moya C., Larrea L., Guerreiro M. (2024). Development and characterization of a cell donor registry for virus-specific T cell manufacture in a blood bank. HLA.

