# Supplementary material for: A High-Throughput Sequencing Strategy for Clinical Repertoire Profiling of T Cell Receptor Beta Chain: Development and Reference Values Across Healthy Adults, Paediatrics, and Cord Blood Units
Source: Int J Mol Sci. 2025 Oct 1;26(19):9590. doi: 10.3390/ijms26199590 (PMC12525236; doi:10.3390/ijms26199590)
Supplement: Supplementary file 1 [file ijms-26-09590-s001.zip › Supplementary Figures rev1.pdf]

Figure S1

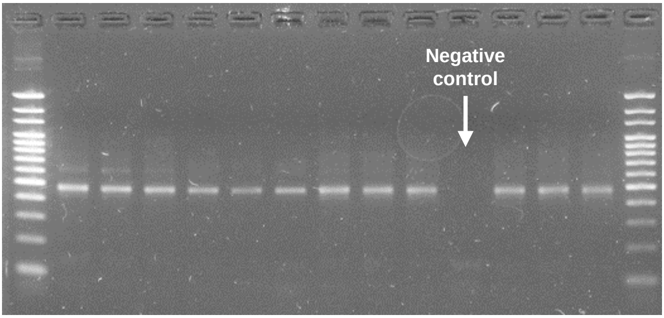

Supplementary figure 1. Agarose gel visualization of the amplified and indexed product. Each well contains libraries of a single sample, in which a band of approximately 480bp is observed. In the well indicated as a negative control, the DNA was replaced with water prior to amplification.

Figure S2

1. Experimental design

- Low resolution → 10,000 – 50,000 T cells (~5 reads/T cell)
  (i.e. clonal repertoires, in vitro expanded T cells, interest on top clonotypes)
- High resolution → ≥ 50,000 T cells (~10 reads/T cell)
  (i.e. diverse repertoires, in-depth study of the T cell repertoire, interest on low-frequency clonotypes)

Sample	gDNA concentration	% T cells	Total ng / replicate[1]	T cell ng / replicate	Number of replicates	Targeted T cells[2]	Sample coverage (reads)
1	70 ng/µl	100%	350 ng	350 ng	1	~50,000	~500,000
2	70 ng/µl	50%	350 ng	175 ng	2	~50,000	~500,000

[1] 5 µl DNA / PCR replicate; [2] Weight of a diploid genome: ~6.6 pg

2. Sample preparation and first PCR - TRB specific amplification

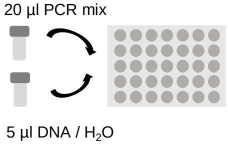

Instruments required	Processing time
DNA spectrophotometer<br>Laminar flow cabinet<br>Veriti Thermal cycler	Hands-on time: ~ 2 hour 30 minutes<br>PCR time: 1 hour 45 minutes<br>Total time: ~ 4 hours 15 minutes

3. Second PCR - library preparation

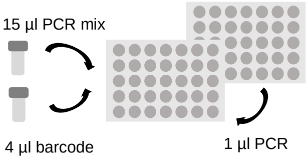

Instruments required	Processing time
Laminar flow cabinet<br>Veriti Thermal cycler	Hands-on time: ~ 1 hour<br>PCR time: 45 minutes<br>Total time: ~ 1 hours 45 minutes

4. Visualization, quantification and pooling

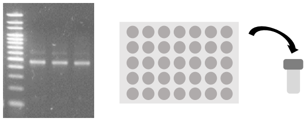

Instruments required	Processing time
Gel electrophoresis system<br>Qubit 4 Fluorometer	Hands-on time: ~ 2 hour 30 minutes<br>Gel electrophoresis: 45 minutes<br>Total time: ~ 3 hours 15 minutes

5. Purification

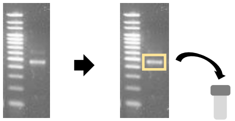

Instruments required	Processing time
Gel electrophoresis system<br>Thermoblock<br>Qubit 4 Fluorometer	Hands-on time: ~ 1 hour 30 minutes<br>Gel electrophoresis: 1 hour 20 minutes<br>Total time: ~ 2 hours 50 minutes

6. Next Generation Sequencing

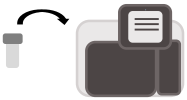

Instruments required	Processing time
Qubit 4 Fluorometer<br>Illumina MiSeq System	Hands-on time: ~2 hour<br>Sequencing: 40 hour<br>Total time: ~ 42 hours

7. Data analysis and sample report

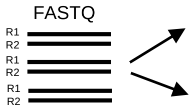

QIAGEN CLC Genomics Workbench / MIXCR
(synthetic DNA controls)

MIXCR / VIDJIL / VDJtools
(DNA samples, negative controls)

Total time: ~ 1 hour / sample

Supplementary figure 2. Workflow of the developed strategy, from sample preparation to data analysis. Instruments and processing times are indicated for each step. The T-cell numbers and sequencing depth indicated in the experimental design are proposed based on our laboratory's experience. Sequencing outputs (FASTQ files) are analyzed using dedicated software. While VIDJIL was not applied in this study, it has been successfully used in our laboratory to process data generated with this strategy.

Figure S3

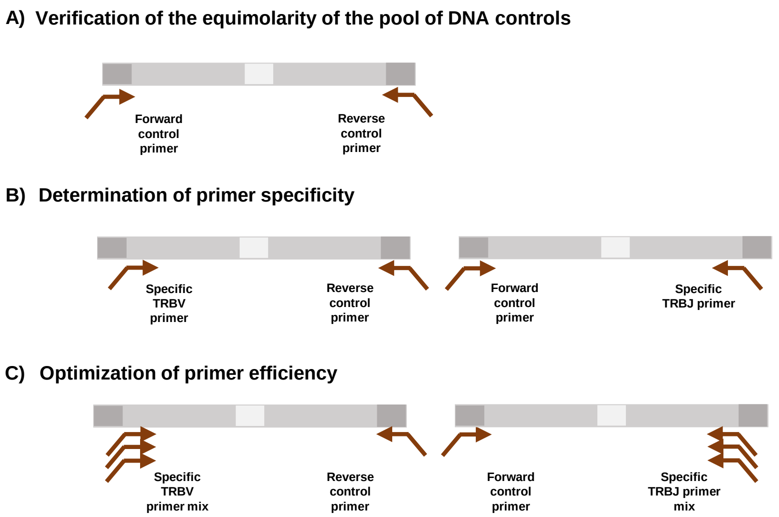

Supplementary figure 3. Optimization of primer specificity and efficiency. A) Amplification of control DNA fragments with the control primer pair to verify the equimolarity of the fragment pool. **B)** Amplification of control DNA fragments with a combination of a control primer and a TRB primer to determine the specificity of each designed primer. **C)** Amplification of control DNA fragments with TRBV and TRBJ primer mixes and control primers to determine and optimize the efficiency of each primer within the mixture.

Figure S4

A)

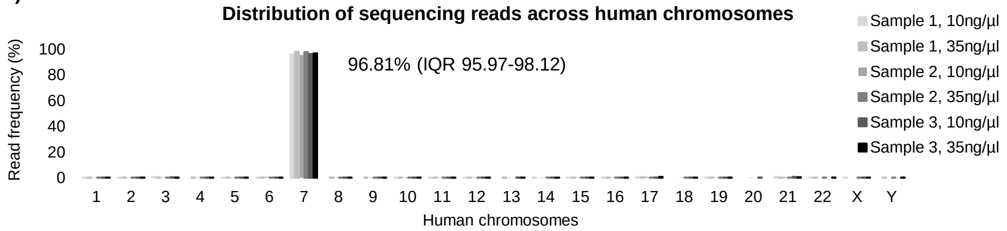

B)

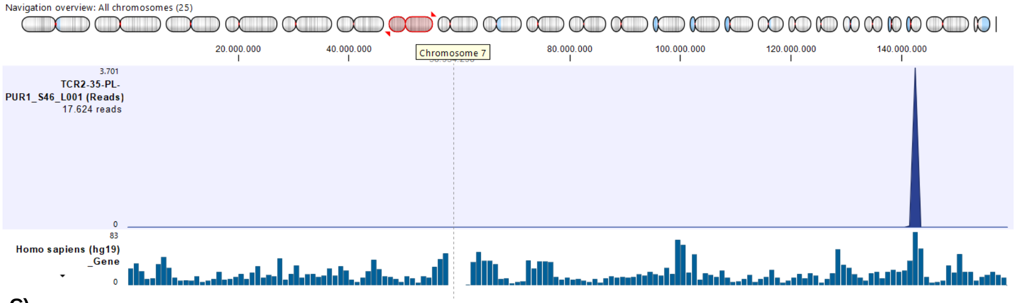

C)

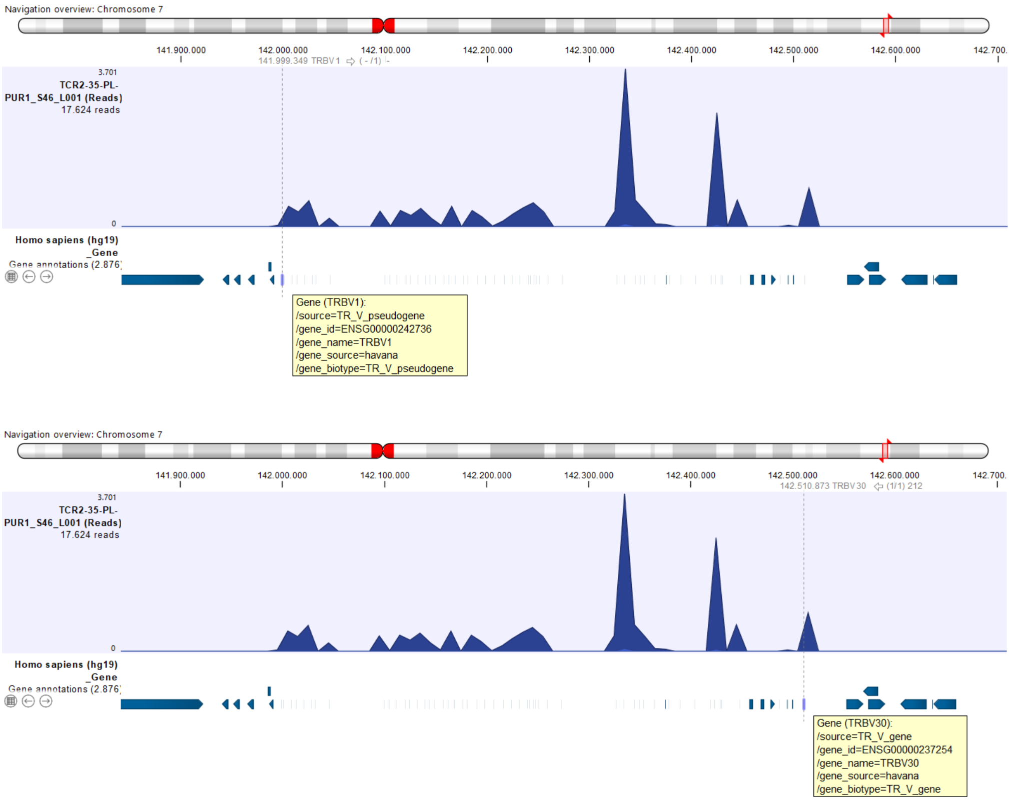

Supplementary figure 4. Specificity of sequencing reads to the human TRB locus. A) Distribution of sequencing reads across human chromosomes in three peripheral blood samples at two starting DNA concentration (35 and 10 ng/µl). Median and IQR values for chromosome 7 alignment are presented. **B)** Distribution of sequencing reads across human chromosome 7 (sample 1, 35 ng/µl). **C)** Zoom-in of the read peak observed in panel B. The reads align exclusively within the TRB locus, spanning from the TRBV1 gene to TRBV30. Alignment performed using the QIAGEN CLC Genomics Workbench (Qiagen).

Figure S5

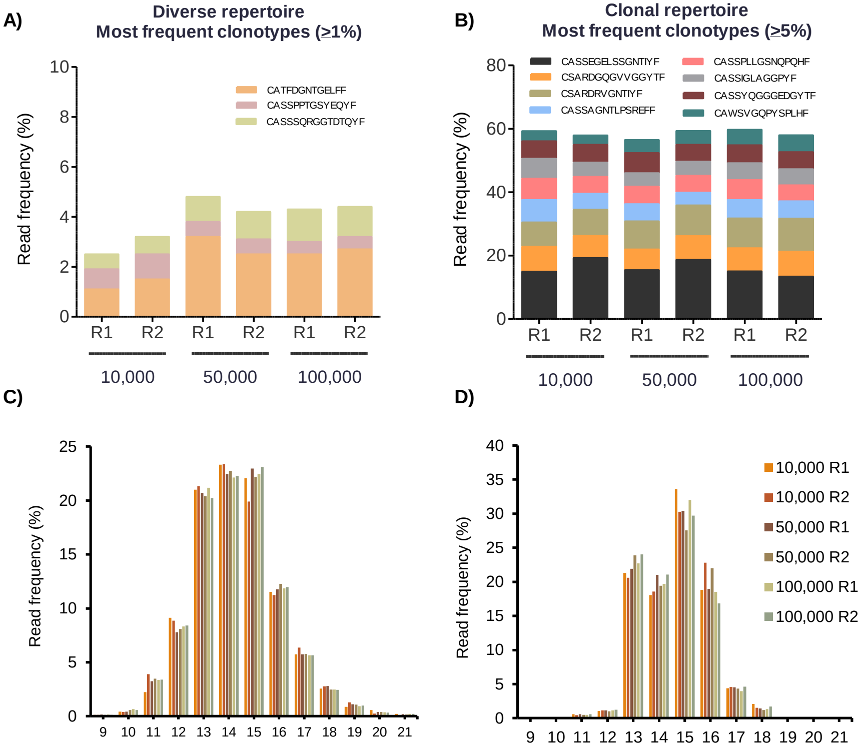
A)
Diverse repertoire
Most frequent clonotypes (≥1%)
Read frequency (%)
CATFDGNTGELFF
CASSPPTGSYEQYF
CASSSQRGGTDTQYF
R1 R2 R1 R2 R1 R2
10,000 50,000 100,000
B)
Clonal repertoire
Most frequent clonotypes (≥5%)
Read frequency (%)
CASSEGELSSGNTIYF
CSARDGQGVVGGYTF
CSARDRVGNTIYF
CASSAGNTLPSREFF
CASSPLLGSNQPQHF
CASSIGLAGGPYF
CASSYQGGGEDGYTF
CAWSVGQPYSPLHF
R1 R2 R1 R2 R1 R2
10,000 50,000 100,000
C)
Read frequency (%)
9 10 11 12 13 14 15 16 17 18 19 20 21
D)
Read frequency (%)
10,000 R1
10,000 R2
50,000 R1
50,000 R2
100,000 R1
100,000 R2
9 10 11 12 13 14 15 16 17 18 19 20 21

Figure S5 (continued)

E)

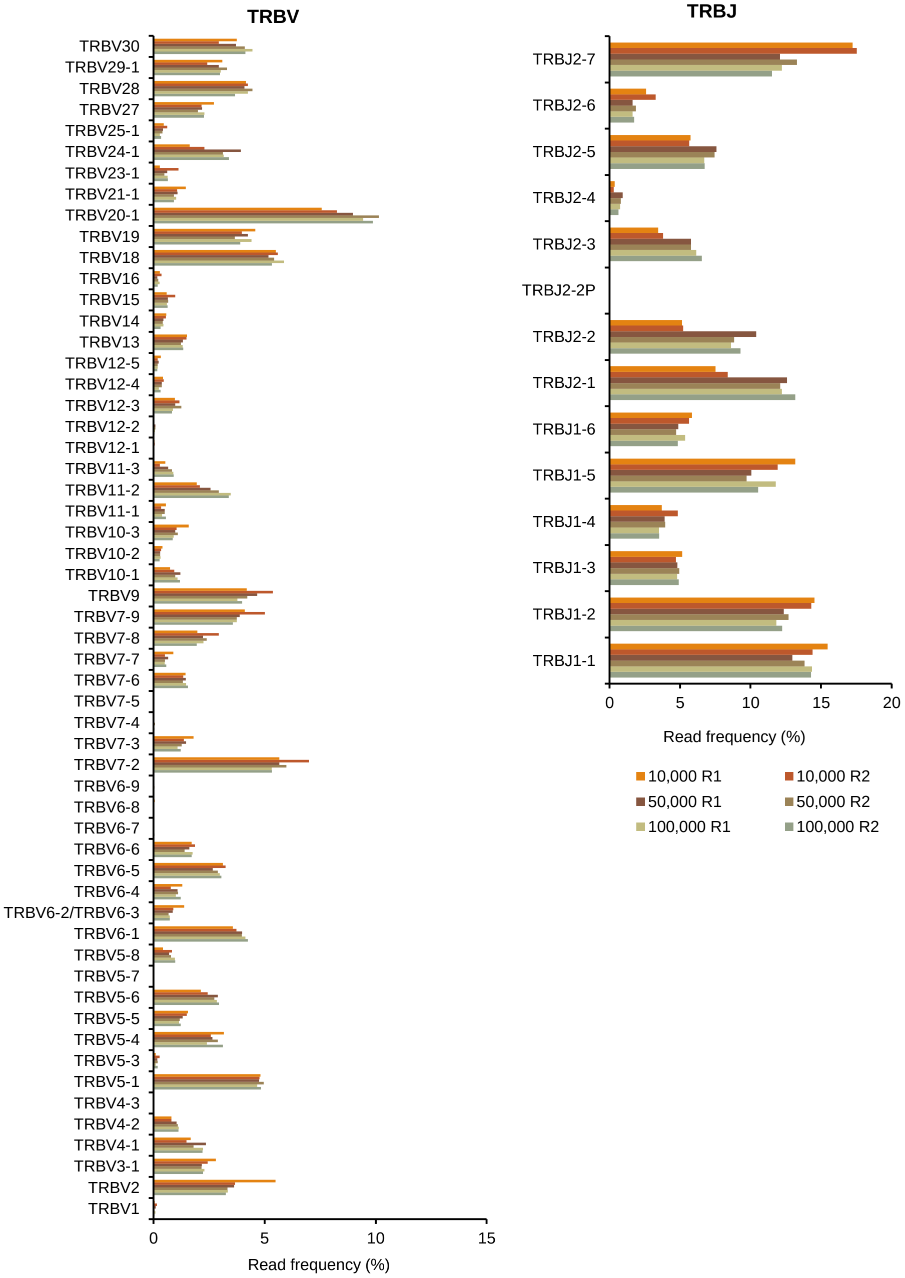

Figure S5 (continued)

F)

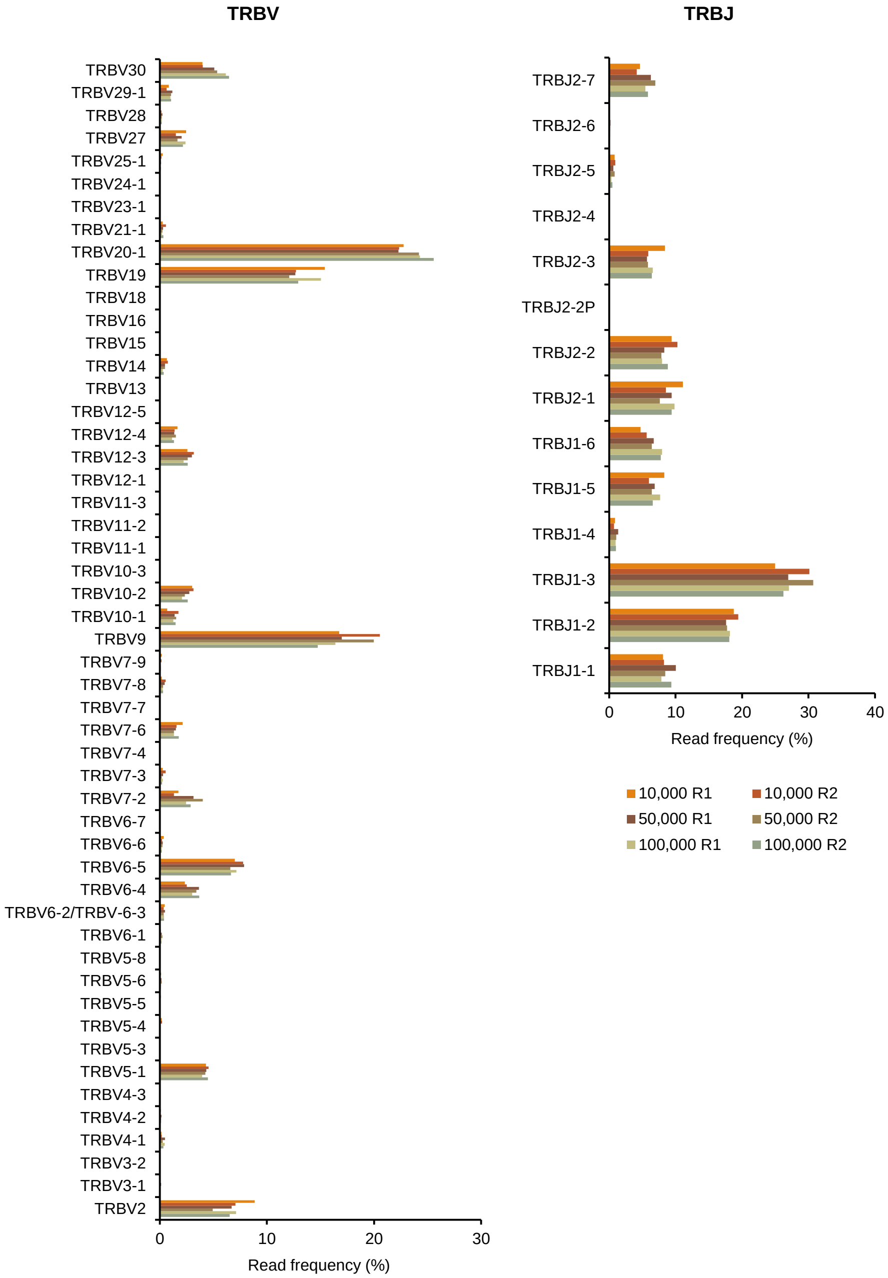

Supplementary figure 5. Comparative analysis of the most frequent clonotypes, CDR3 length distribution and TRB segment usage between replicates with different amounts of T cells. A) Read frequency of the main clonotypes detected in two replicates (R1 and R2) under the different conditions tested (10,000, 50,000 and 100,000 T cells) of the diverse repertoire. Main clonotypes were considered those with a frequency $\geq$1%. **B)** Read frequency of the main clonotypes detected in two replicates (R1 and R2) under the different conditions tested (10,000, 50,000 and 100,000 T cells) of the clonal repertoire. Main clonotypes were considered those with a frequency $\geq$5%. **C)** CDR3 length distribution in two replicates (R1 and R2) under the different conditions tested (10,000, 50,000 and 100,000 T cells) of the diverse repertoire. **D)** CDR3 length distribution in two replicates (R1 and R2) under the different conditions tested (10,000, 50,000 and 100,000 T cells) of the clonal repertoire. **E)** Use of TRB segments in two replicates (R1 and R2) under the different conditions tested (10,000, 50,000 and 100,000 T cells) of the diverse repertoire. **F)** Use of TRB segments in two replicates (R1 and R2) under the different conditions tested (10,000, 50,000 and 100,000 T cells) of the clonal repertoire.

Figure S6

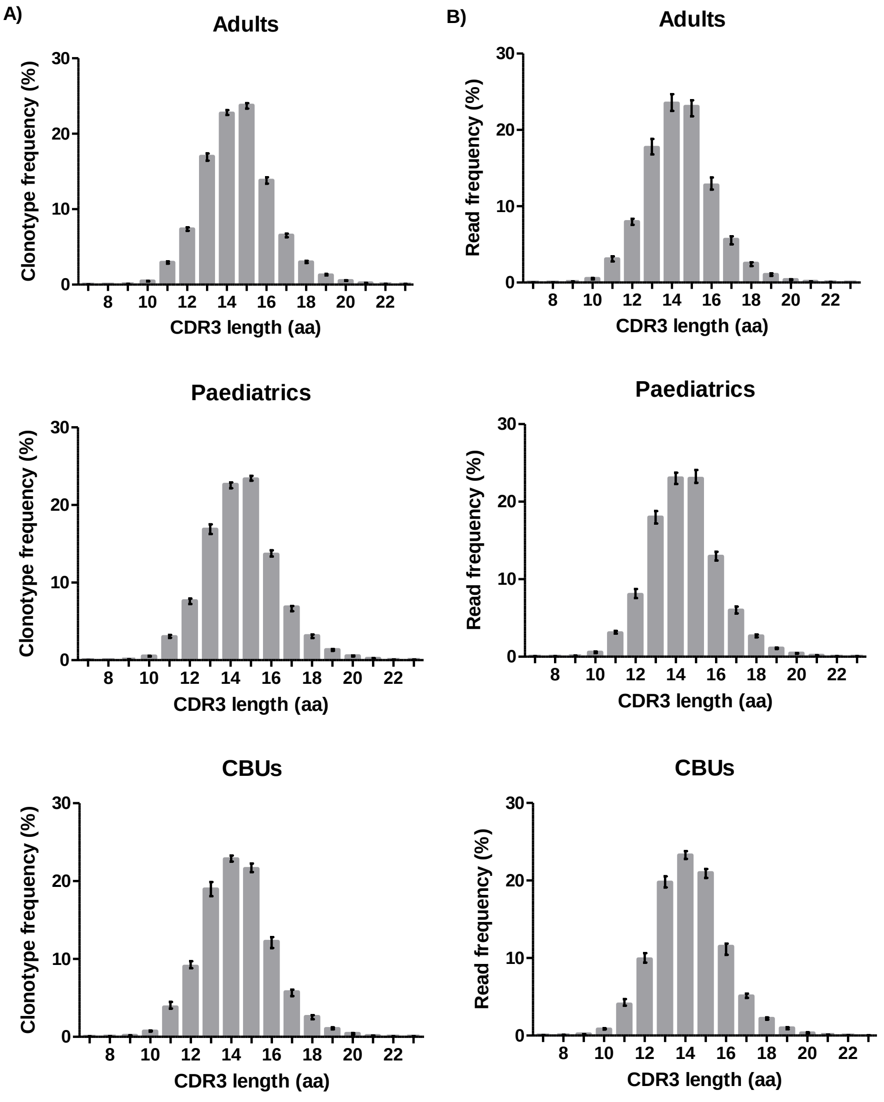

Supplementary figure 6. CDR3 length distribution in adults, paediatrics and cord blood units. A) Distribution of CDR3 amino acid length in adults, paediatrics and CBUs, calculated from unique clonotypes. Bars represent the median and error bars represent the interquartile range. **B)** Distribution of CDR3 amino acid length in adults, paediatrics and CBUs, calculated from total sequences. Bars represent the median and error bars represent the interquartile range. aa, amino acid; CBU, cord blood unit.

Figure S7

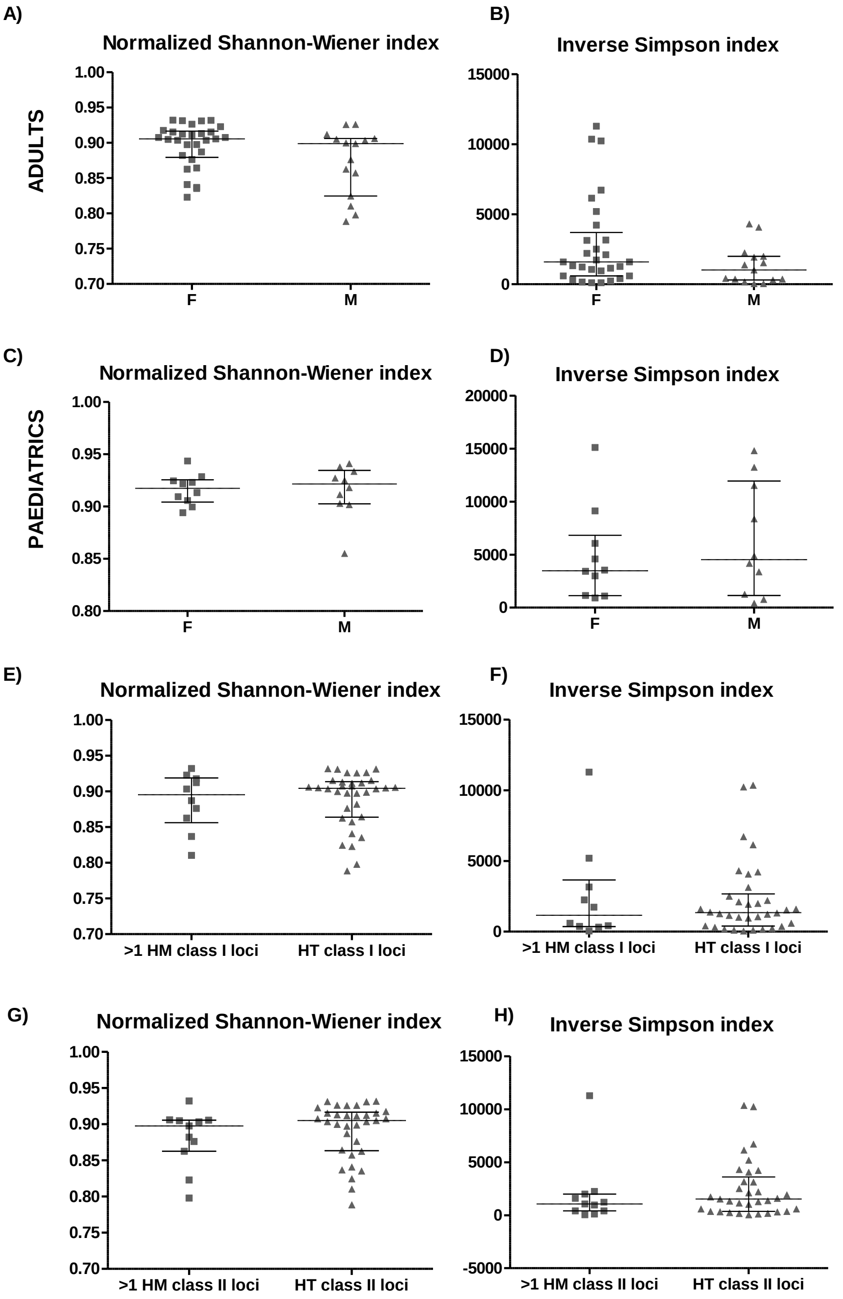
A)
Normalized Shannon-Wiener index
ADULTS
1.00
0.95
0.90
0.85
0.80
0.75
0.70
F
M
B)
Inverse Simpson index
15000
10000
5000
0
F
M
C)
Normalized Shannon-Wiener index
PAEDIATRICS
1.00
0.95
0.90
0.85
0.80
F
M
D)
Inverse Simpson index
20000
15000
10000
5000
0
F
M
E)
Normalized Shannon-Wiener index
1.00
0.95
0.90
0.85
0.80
0.75
0.70
>1 HM class I loci
HT class I loci
F)
Inverse Simpson index
15000
10000
5000
0
>1 HM class I loci
HT class I loci
G)
Normalized Shannon-Wiener index
1.00
0.95
0.90
0.85
0.80
0.75
0.70
>1 HM class II loci
HT class II loci
H)
Inverse Simpson index
15000
10000
5000
0
-5000
>1 HM class II loci
HT class II loci

Supplementary figure 7. Diversity metrics and sex, HLA class I and HLA class II polymorphisms. A) Normalized Shannon-Wiener index according to sex in adult donors. **B)** Inverse Simpson index according to sex in adults donors. **C)** Normalized Shannon-Wiener index according to sex in paediatric donors. **D)** Inverse Simpson index according to sex in paediatric donors. **E)** Normalized Shannon-Wiener index in adult donors with $\geq$ 1 homozygous HLA class I loci or in donors with all HLA class I heterozygous. Class I loci analysed: HLA-A, -B and –C. **F)** Inverse Simpson index in adult donors with $\geq$ 1 homozygous HLA class I loci or in donors with all HLA class I heterozygous. Class I loci analysed: HLA-A, -B and –C. **G)** Normalized Shannon-Wiener index in adult donors with $\geq$ 1 homozygous HLA class II loci or in donors with all HLA class II heterozygous. Class II loci analysed: HLA-DRB1 and -DQB1. **H)** Inverse Simpson index in adult donors with $\geq$ 1 homozygous HLA class II loci or in donors with all HLA class II heterozygous. Class II loci analysed: HLA-DRB1 and -DQB1. Error bars in scatter dot plots represent the median and interquartile range. Data were analysed by Mann Whitney test. F, female; HM, homozygous; HT, heterozygous; M, male.

Figure S8

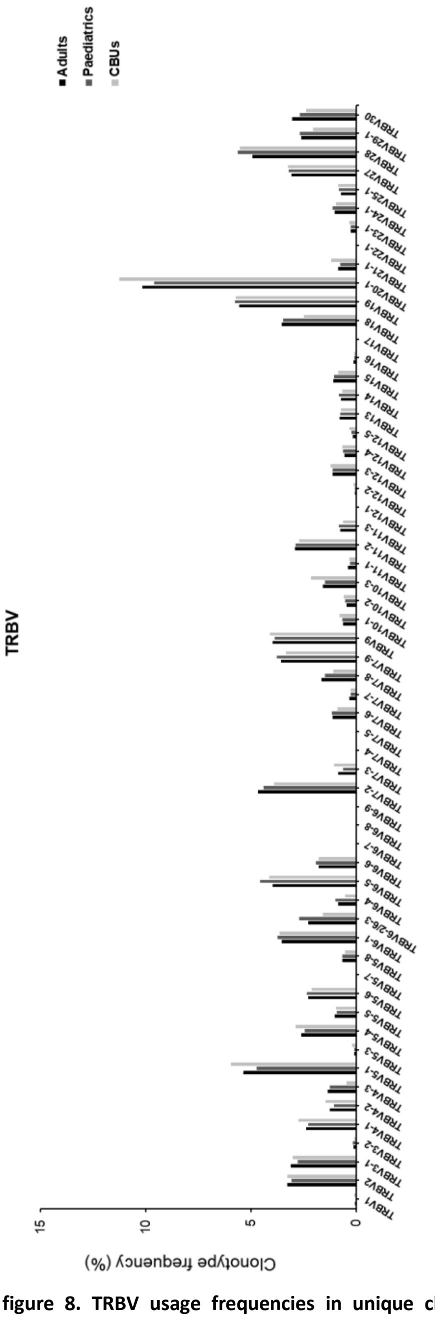

Supplementary figure 8. TRBV usage frequencies in unique clonotypes. Median frequency of clonotypes with each TRBV gene considering the total number of unique clonotypes in adult, paediatric, and CBU donors. CBU, cord blood unit.

Figure S9

A)

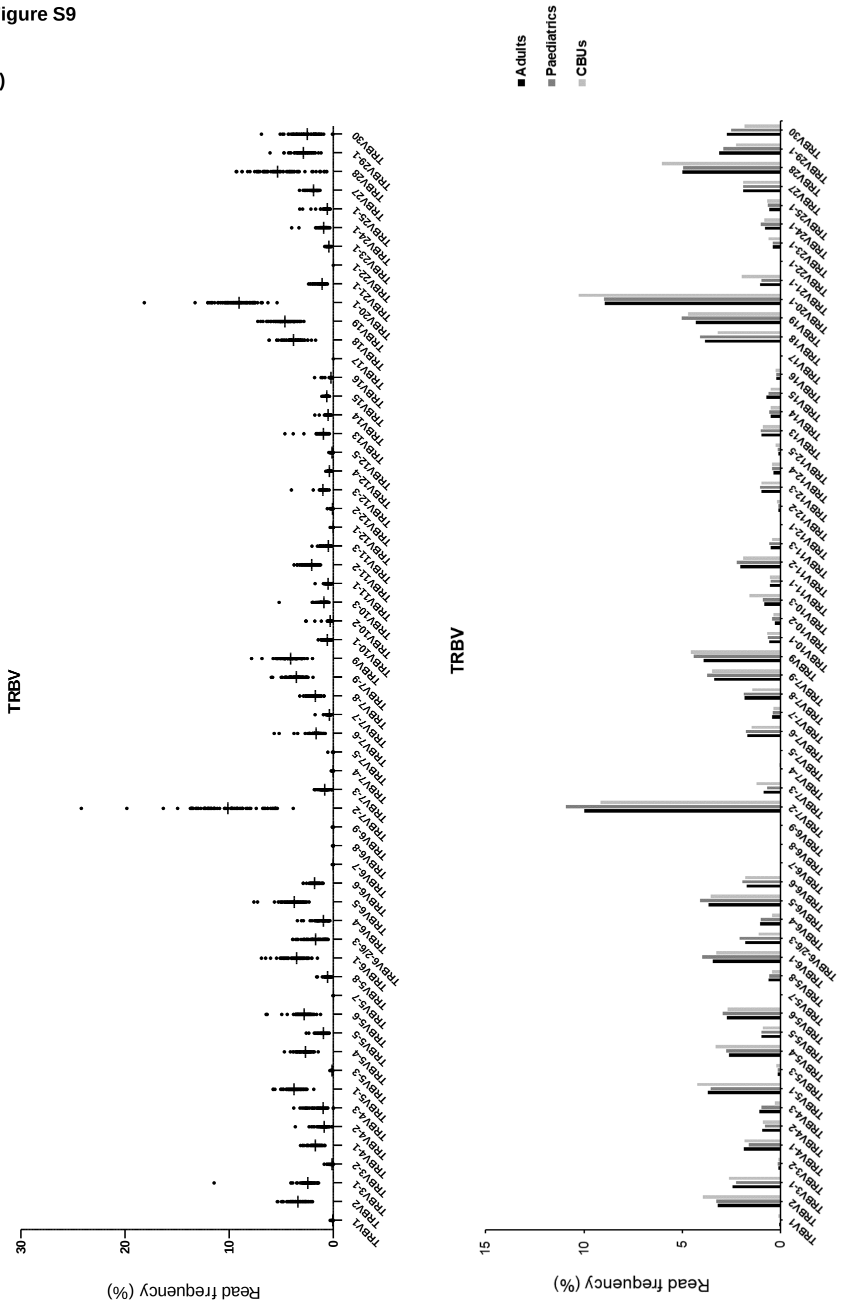

Supplementary figure 9. TRBV usage frequencies considering read frequency of each unique clonotype. A) Read frequency of clonotypes with each TRBV gene considering the total number of reads in all samples. **B)** Read frequency of clonotypes with each TRBV gene considering the total number of reads in all samples in adult, paediatric, and CBU donors. The horizontal line and bars represent the median frequency. CBU, cord blood unit.

Figure S10

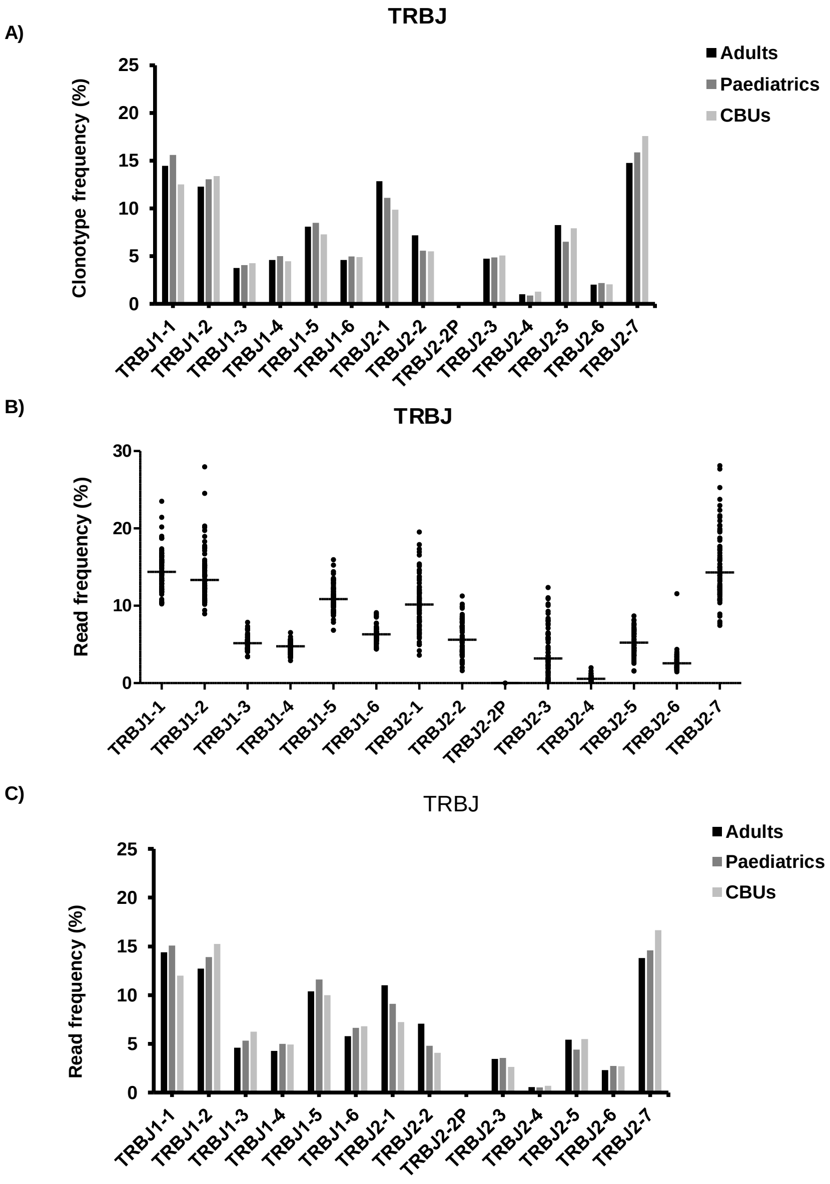

Supplementary figure 10. TRBJ usage frequencies. A) Median frequency of clonotypes with each TRBJ gene considering the total number of unique clonotypes in adult, paediatric and CBU donors. **B)** Read frequency of clonotypes with each TRBJ gene considering the total number of reads in all samples. **C)** Read frequency of clonotypes with each TRBJ gene considering the total number of reads in adult, paediatric, and CBU donors. Horizontal line and bars represent the median frequency. CBU, cord blood unit.

Figure S11

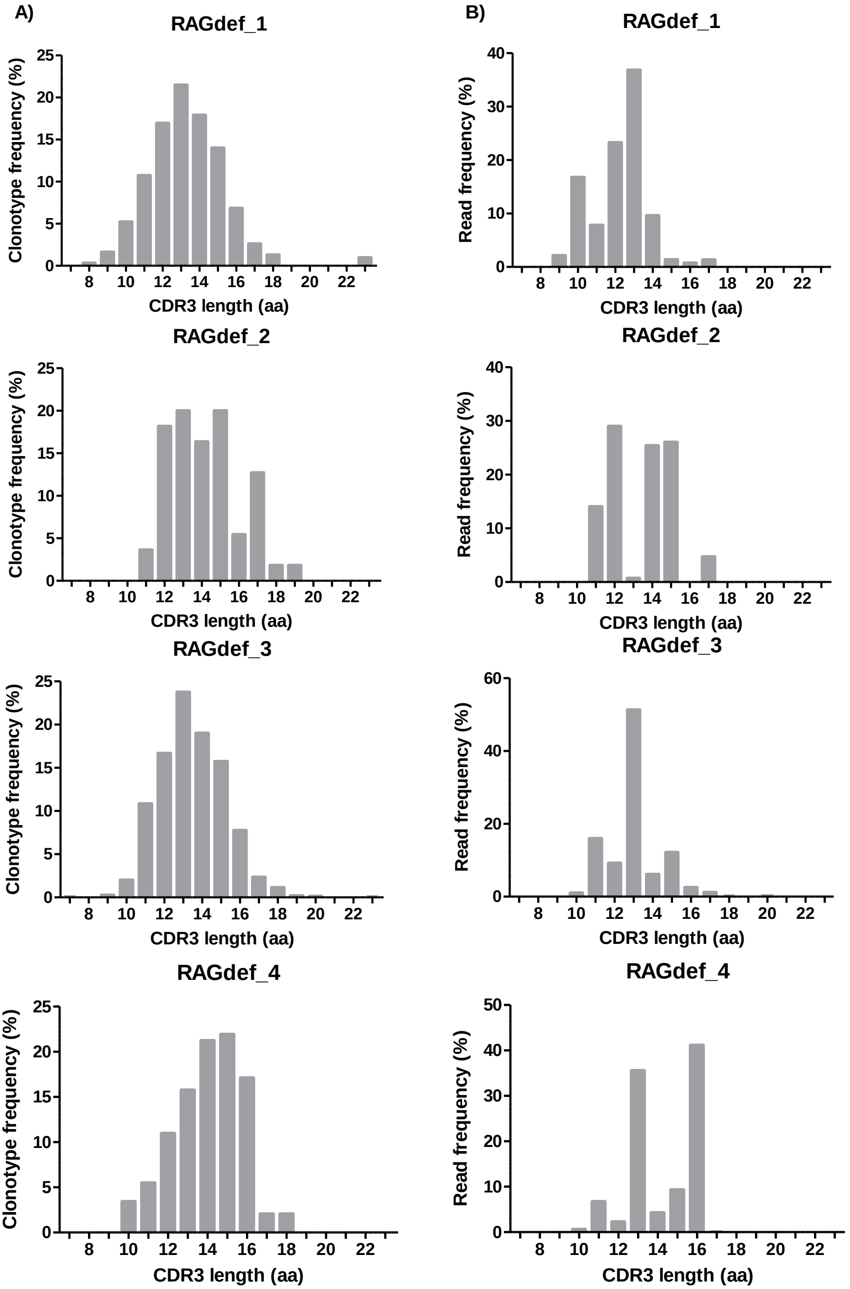

Supplementary figure 11. CDR3 length distribution in patients with RAG-SCID/CID. A) Distribution of CDR3 amino acid length in patients with RAG-SCID/CID calculated from unique clonotypes. **B)** Distribution of CDR3 amino acid length in patients with RAG-SCID/CID calculated from total sequences. Aa, aminoacids; def, deficiency; RAG, recombination activating genes; SCID/CID, Severe combined immunodeficiency / Combined immunodeficiency.

Figure S12

A)

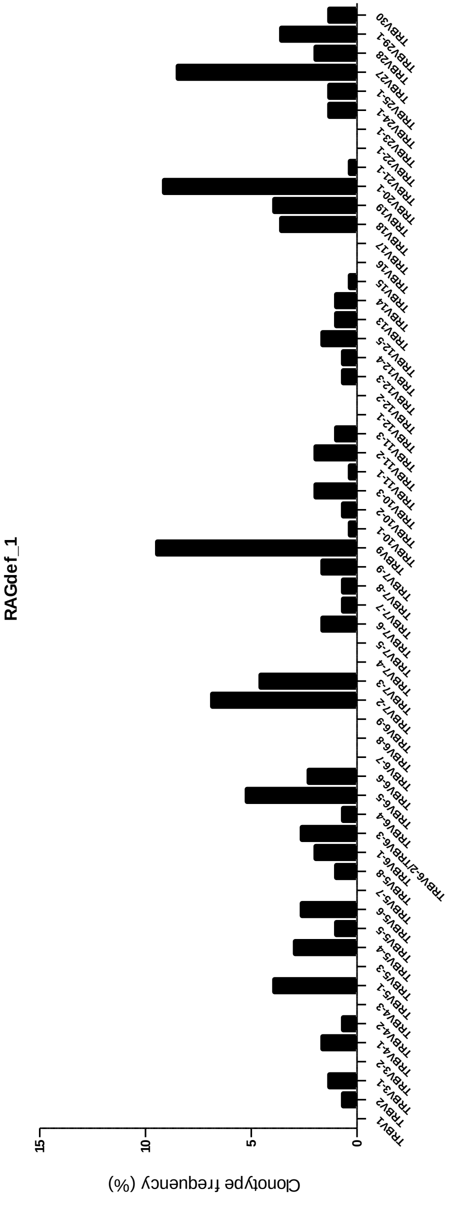
RAGdef_1
Clonotype frequency (%)
0
5
10
15
TRBV1
TRBV2
TRBV3-1
TRBV3-2
TRBV4-1
TRBV4-2
TRBV4-3
TRBV5-1
TRBV5-3
TRBV5-4
TRBV5-5
TRBV5-6
TRBV5-7
TRBV5-8
TRBV6-2/TRBV6-1
TRBV6-3
TRBV6-4
TRBV6-5
TRBV6-6
TRBV6-7
TRBV6-8
TRBV6-9
TRBV7-2
TRBV7-3
TRBV7-4
TRBV7-5
TRBV7-6
TRBV7-7
TRBV7-8
TRBV7-9
TRBV9
TRBV10-1
TRBV10-2
TRBV10-3
TRBV11-1
TRBV11-2
TRBV11-3
TRBV12-1
TRBV12-2
TRBV12-3
TRBV12-4
TRBV12-5
TRBV13
TRBV14
TRBV15
TRBV16
TRBV17
TRBV18
TRBV19
TRBV20-1
TRBV21-1
TRBV22-1
TRBV23-1
TRBV24-1
TRBV25-1
TRBV27
TRBV28
TRBV29-1
TRBV30

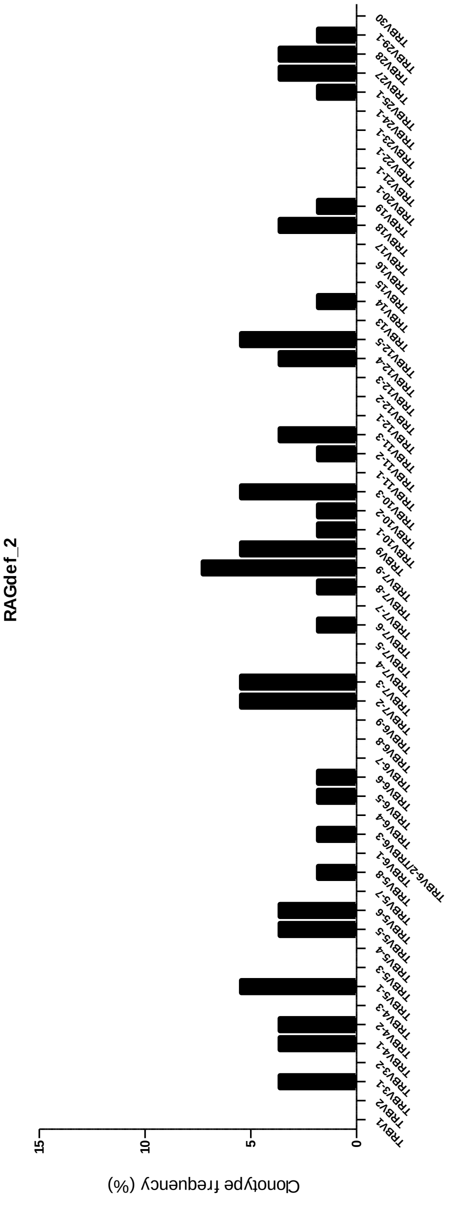
RAGdef_2
Clonotype frequency (%)
0
5
10
15
TRBV1
TRBV2
TRBV3-1
TRBV3-2
TRBV4-1
TRBV4-2
TRBV4-3
TRBV5-1
TRBV5-3
TRBV5-4
TRBV5-5
TRBV5-6
TRBV5-7
TRBV5-8
TRBV6-2/TRBV6-1
TRBV6-3
TRBV6-4
TRBV6-5
TRBV6-6
TRBV6-7
TRBV6-8
TRBV6-9
TRBV7-2
TRBV7-3
TRBV7-4
TRBV7-5
TRBV7-6
TRBV7-7
TRBV7-8
TRBV7-9
TRBV9
TRBV10-1
TRBV10-2
TRBV10-3
TRBV11-1
TRBV11-2
TRBV11-3
TRBV12-1
TRBV12-2
TRBV12-3
TRBV12-4
TRBV12-5
TRBV13
TRBV14
TRBV15
TRBV16
TRBV17
TRBV18
TRBV19
TRBV20-1
TRBV21-1
TRBV22-1
TRBV23-1
TRBV24-1
TRBV25-1
TRBV27
TRBV28
TRBV29-1
TRBV30

Figure S12 (continued)

A)

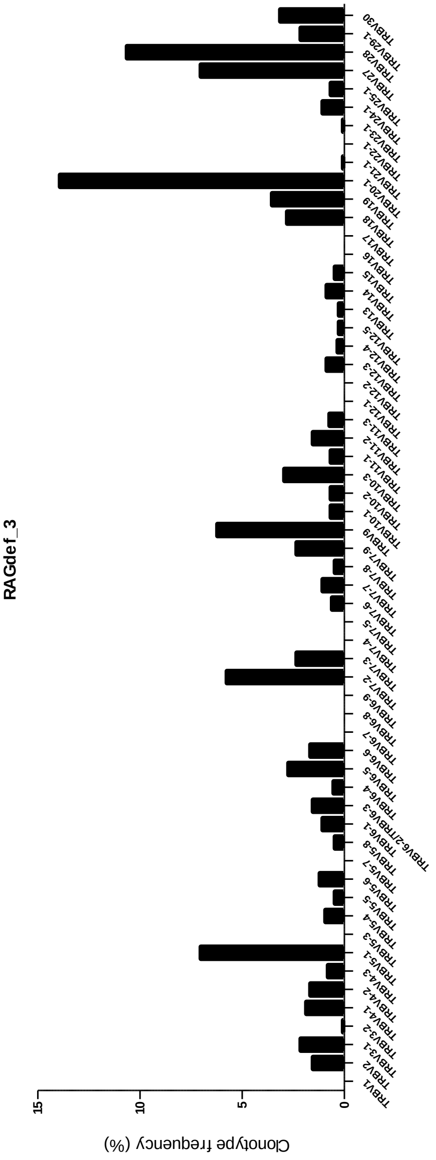

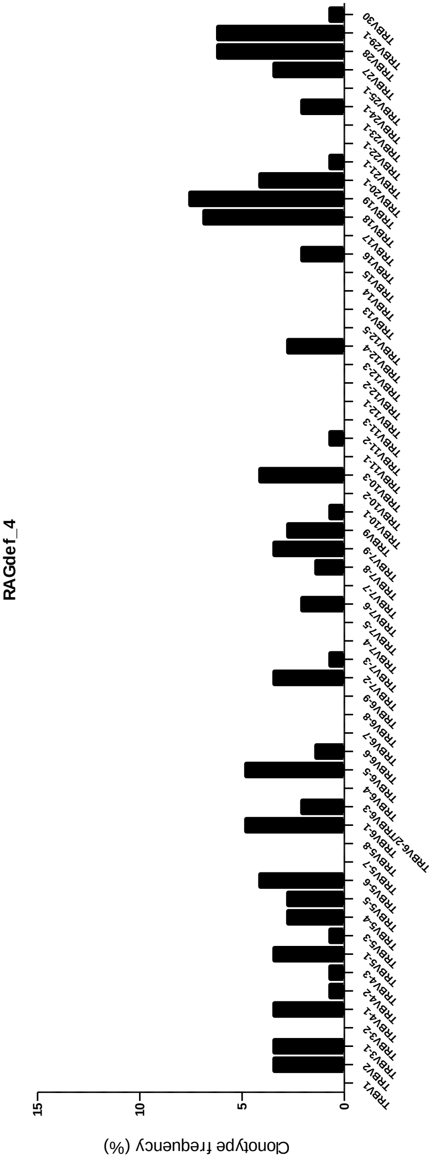

Figure S12 (continued)

B)

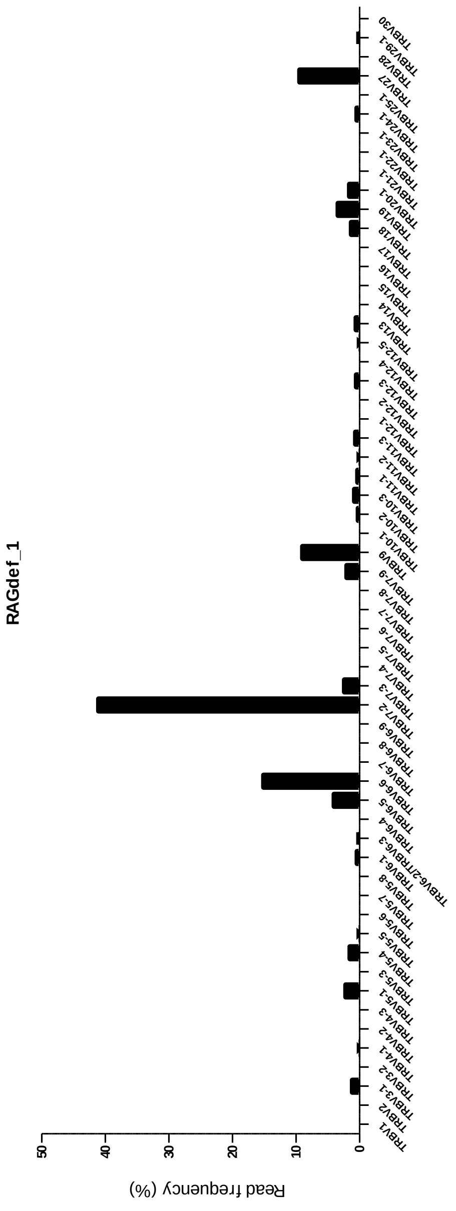

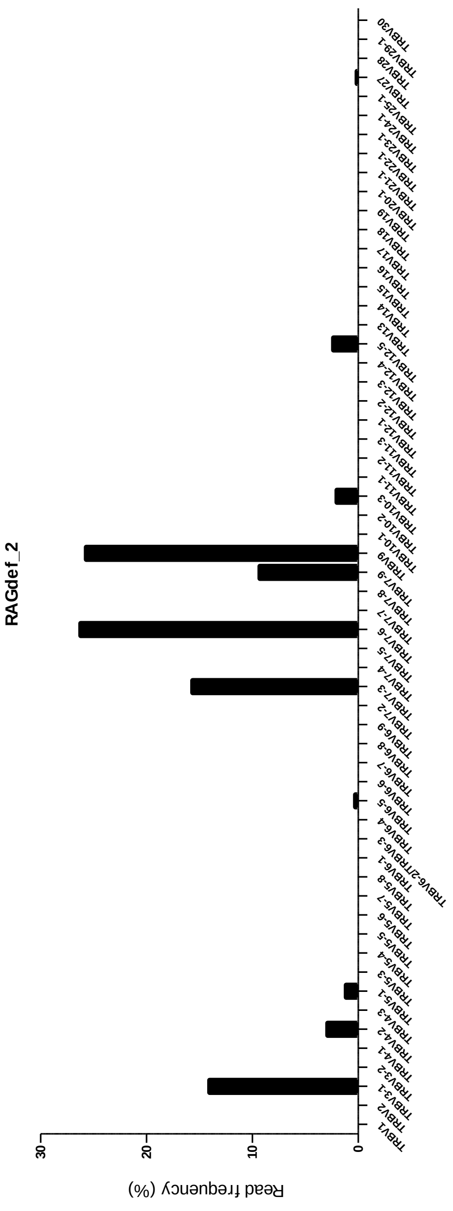

Figure S12 (continued)

B)

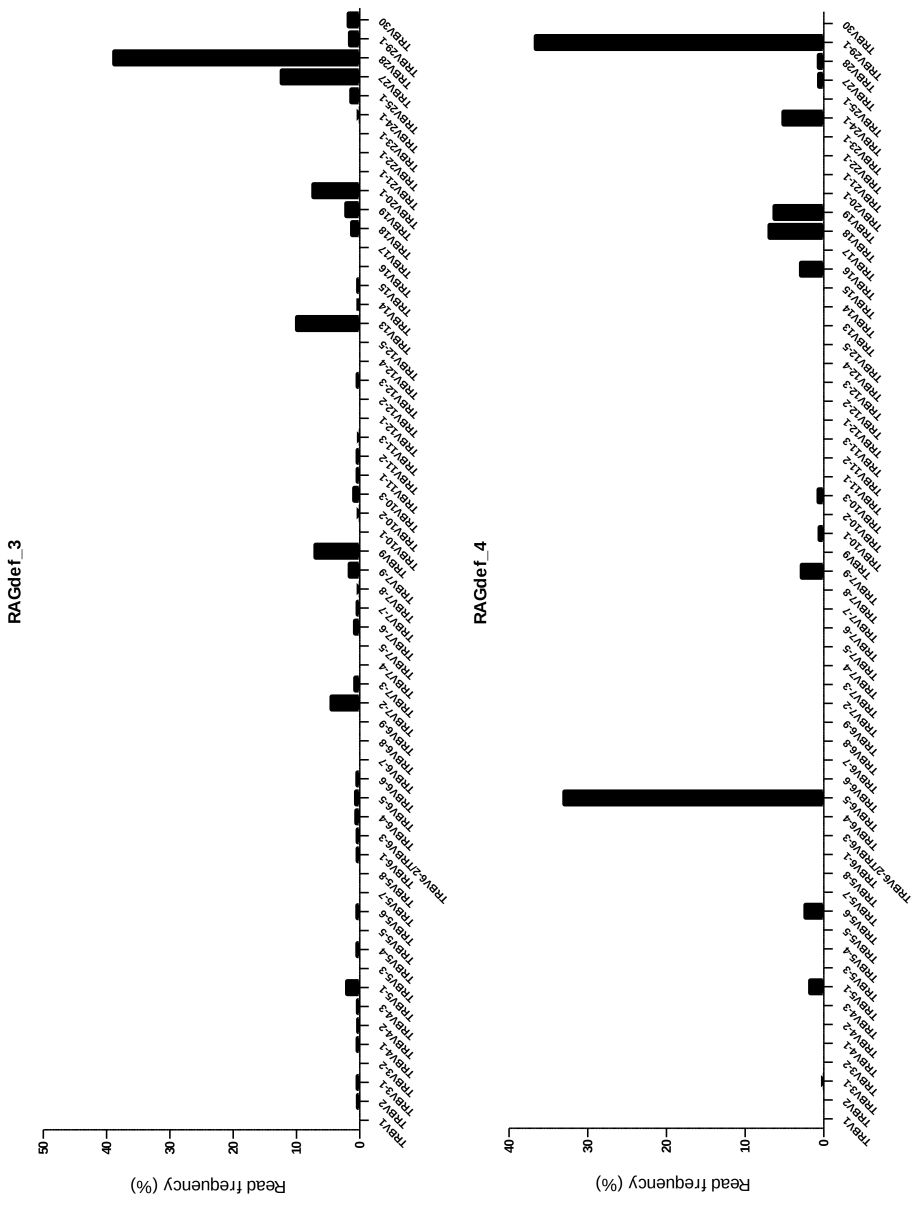

Figure S12 (continued)

C)

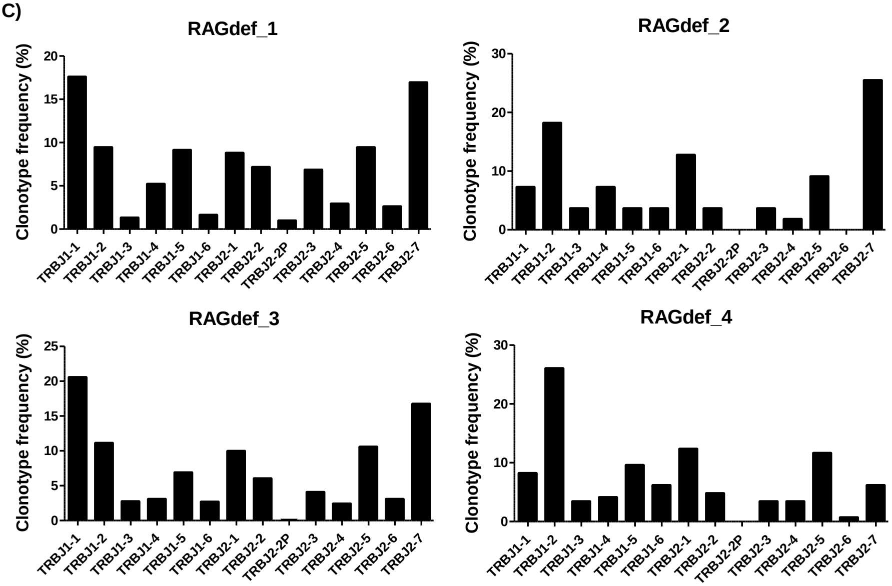

D)

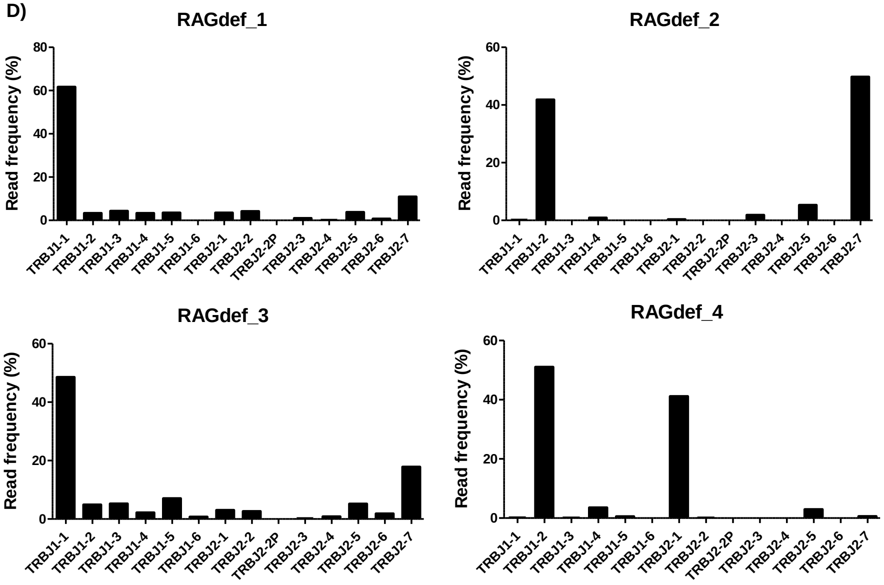

Supplementary figure 12. TRBV and TRBJ usage frequencies in patients with RAG-SCID/CID. A) Frequency of unique clonotypes with each TRBV gene considering the total number of unique clonotypes. **B)** Read frequency of clonotypes with each TRBV gene considering the total number of reads. **C)** Frequency of unique clonotypes with each TRBJ gene considering the total number of unique clonotypes. **D)** Read frequency of clonotypes with each TRBJ gene considering the total number of reads. Def, deficiency; RAG, recombination activating genes; SCID/CID, Severe combined immunodeficiency / Combined immunodeficiency.
